# Phenotypic Adaptation to Antiseptics and Effects on Biofilm Formation Capacity and Antibiotic Resistance in Clinical Isolates of Early Colonizers in Dental Plaque

**DOI:** 10.3390/antibiotics11050688

**Published:** 2022-05-19

**Authors:** David L. Auer, Xiaojun Mao, Annette Carola Anderson, Denise Muehler, Annette Wittmer, Christiane von Ohle, Diana Wolff, Cornelia Frese, Karl-Anton Hiller, Tim Maisch, Wolfgang Buchalla, Elmar Hellwig, Ali Al-Ahmad, Fabian Cieplik

**Affiliations:** 1 Department of Conservative Dentistry and Periodontology, University Hospital Regensburg, 93053 Regensburg, Germany; david.auer@ukr.de (D.L.A.); olivermxj@sina.com (X.M.); denise.muehler@ukr.de (D.M.); karl-anton.hiller@ukr.de (K.-A.H.); wolfgang.buchalla@ukr.de (W.B.); 2 Department of Operative Dentistry and Periodontology, Center for Dental Medicine, Faculty of Medicine, University of Freiburg, 79085 Freiburg im Breisgau, Germany; annette.anderson@uniklinik-freiburg.de (A.C.A.); elmar.hellwig@uniklinik-freiburg.de (E.H.); ali.al-ahmad@uniklinik-freiburg.de (A.A.-A.); 3 Institute of Medical Microbiology and Hygiene, Faculty of Medicine, University of Freiburg, 79085 Freiburg im Breisgau, Germany; annette.wittmer@uniklinik-freiburg.de; 4 Department of Conservative Dentistry, Periodontology and Endodontology, University Hospital Tübingen, 72076 Tübingen, Germany; christiane.von_ohle@med.uni-tuebingen.de; 5 Department of Conservative Dentistry, University Hospital Heidelberg, 69120 Heidelberg, Germany; diana.wolff@med.uni-heidelberg.de (D.W.); cornelia.frese@med.uni-heidelberg.de (C.F.); 6 Department of Dermatology, University Hospital Regensburg, 93053 Regensburg, Germany; tim.maisch@ukr.de

**Keywords:** chlorhexidine, cetylpyridinium chloride, antiseptic, biocide, resistance, adaptation, oral biofilm, antibiotic

## Abstract

Despite the wide-spread use of antiseptics in dental practice and oral care products, there is little public awareness of potential risks associated with antiseptic resistance and potentially concomitant cross-resistance. Therefore, the aim of this study was to investigate potential phenotypic adaptation in 177 clinical isolates of early colonizers of dental plaque (*Streptococcus*, *Actinomyces*, *Rothia* and *Veillonella* spp.) upon repeated exposure to subinhibitory concentrations of chlorhexidine digluconate (CHX) or cetylpyridinium chloride (CPC) over 10 passages using a modified microdilution method. Stability of phenotypic adaptation was re-evaluated after culture in antiseptic-free nutrient broth for 24 or 72 h. Strains showing 8-fold minimal inhibitory concentration (MIC)-increase were further examined regarding their biofilm formation capacity, phenotypic antibiotic resistance and presence of antibiotic resistance genes (ARGs). Eight-fold MIC-increases to CHX were detected in four *Streptococcus* isolates. These strains mostly exhibited significantly increased biofilm formation capacity compared to their respective wild-type strains. Phenotypic antibiotic resistance was detected to tetracycline and erythromycin, consistent with the detected ARGs. In conclusion, this study shows that clinical isolates of early colonizers of dental plaque can phenotypically adapt toward antiseptics such as CHX upon repeated exposure. The underlying mechanisms at genomic and transcriptomic levels need to be investigated in future studies.

## 1. Introduction

Bacterial antimicrobial resistance (AMR) has emerged as one of the greatest public health threats and challenges of the 21st century [1]. The Review on Antimicrobial Resistance from 2016 predicted that deaths attributable to AMR could increase to 10 million per year by 2050 if no appropriate counter-action was taken immediately [2]. Only recently, a paper was published in The Lancet, wherein the Antimicrobial Resistance Collaborators group around Christopher Murray estimated median global numbers of 4.95 million deaths associated with bacterial AMR and 1.27 million deaths directly attributable to bacterial AMR in 2019 [3]. Consequently, AMR can be considered the leading cause of death worldwide, trailing only COVID-19 and tuberculosis but ahead of HIV/AIDS and malaria when it comes to deaths from infections [4]. Thus, AMR has been called an overlooked pandemic that continues in the shadows while COVID-19 rages on [4]. In addition, the current increase in disinfection practices and use of antiseptics and biocides due to the COVID-19 pandemic may pose risks by accelerating the spread of AMR [5], as antiseptics such as quaternary ammonium compounds (QACs) [6,7] or chlorhexidine digluconate (CHX) [8,9,10] may foster the spread of AMR by causing selection pressure and mutations and promoting horizontal gene transfer.

Although the oral cavity was highlighted as a potential reservoir for antibiotic resistance genes several years ago [11,12,13], there is little awareness in the dental community of the potential risks associated with the use of antiseptics with regard to AMR [7,8]. This is notable because a wide range of antiseptics, mostly CHX or the QAC cetylpyridinium chloride (CPC), are included in mouthwashes, gels, or toothpastes that are either intended for professional use in the dental office or available as over-the-counter consumer products [14,15,16,17]. For example, since the COVID-19 pandemic, antiseptics have been routinely used as preprocedural mouthwashes to potentially reduce exposure to SARS-CoV-2 and other microorganisms in dental aerosols [18,19,20]. Furthermore, the use of antiseptic mouthwashes has been recommended as adjunct to mechanical biofilm removal and use of fluorides in certain high-risk patient groups such as patients with intellectual disabilities [21], patients undergoing fixed-appliance orthodontic treatment [22] or following surgical procedures [23], elderly people with insufficient manual abilities [24] or people receiving mechanical ventilation aiming to reduce the risk of ventilator-associated pneumonia [25].

As early as in the 1970s, and only a few years after the introduction of CHX into dental practice, several studies reported that long-term clinical use of CHX-containing mouthwashes or gels resulted in the emergence of clinical isolates of *Streptococcus sanguinis* with reduced susceptibility to this antiseptic [26,27,28]. Accordingly, several studies from recent years have shown that laboratory reference strains of oral and non-oral bacteria are able to phenotypically adapt to CHX or CPC upon multiple exposures to sublethal concentrations in vitro [29,30,31]. In this context, the development of cross-resistance to antibiotics has also been reported [9,10]. It is well known that laboratory reference strains may have lost important pathophysiological properties that are only present in clinical isolates [32]. Nevertheless, there has been no report on potential development of resistance to CHX or CPC in clinical oral isolates, to date [7,8,29].

Therefore, the aim of the present study was to investigate the potential phenotypic adaptation to CHX and CPC in clinical isolates of early colonizers of oral biofilm from the genera *Streptococcus*, *Actinomyces*, *Rothia* and *Veillonella*. In addition, the effects of phenotypic adaptation to CHX or CPC on biofilm formation capacity, phenotypic antibiotic resistance, and presence of antibiotic resistance genes were investigated.

## 2. Materials and Methods

### 2.1. Bacterial Strains and Culture Conditions

The 177 clinical isolates used in this study were collected from supragingival plaque samples of healthy and caries-active volunteers who had been recruited in an earlier clinical study approved by the ethical committee of the Universities of Freiburg, Heidelberg and Tübingen (references: 604/16; S-652/2016; 863/201BO2) and registered in the German Clinical Trials Register (DRKS00013119). These 177 bacterial clinical isolates comprised 112 *Streptococcus* spp., 19 *Actinomyces* spp., 20 *Rothia* spp., and 26 *Veillonella* spp. (Table 1).

All isolates were stored at −80 °C in brain heart infusion (BHI; Sigma-Aldrich, St. Louis, MO, USA) broth containing 15% (*v*/*v*) glycerol, as described earlier [33]. Identification of these isolates to the species level was conducted by means of matrix-assisted laser desorption/ionization time-of-flight mass spectrometry (MALDI-TOF MS) employing a Microflex mass spectrometer and BioTyper analysis software (both from Bruker, Billerica, MA, United States) independently in two different laboratories, as described earlier [34,35].

For laboratory use, the frozen bacterial isolates were thawed at 37 °C in a water bath. *Rothia* spp. and *Veillonella* spp. were grown in Schaedler broth (Roth, Karlsruhe, Germany) and on Schaedler agar plates, while *Streptococcus* spp. and *Actinomyces* spp. in BHI broth (Sigma-Aldrich) and on Columbia Agar with sheep blood (all agar plates provided by the Institute for Clinical Microbiology and Hygiene, University Hospital Regensburg, Germany). All isolates were cultured under anaerobic conditions (80% N_2_, 10% CO_2_, and 10% H_2_) in a microincubator (MI23NK, SCHOLZEN Microbiology Systems, St. Margrethen, Switzerland).

### 2.2. Test Substances

Chlorhexidine digluconate (CHX; Sigma C9394) and cetylpyridinium chloride (CPC; Merck 6,002,006; both: Merck, Darmstadt, Germany) were both solved in dH_2_O and diluted to stock solutions (512 µg/mL) for use in the further experiments.

### 2.3. Minimal Inhibitory Concentration (MIC) Passaging and Re-Evaluation of Phenotypic Adaptation

For preparation of planktonic cultures, colonies were picked, suspended in 5 mL of the respective culture broth, and cultured overnight at 37 °C under anaerobic conditions (80% N_2_, 10% CO_2_, and 10% H_2_; microincubator MI23NK) to yield bacteria in the stationary growth phase. For further analyses, cryo banks (Mast Diagnostica Labortechnik, Reinfeld, Germany) were used to store wild-type (WT) cultures at −80 °C.

Two-fold serial dilutions were prepared from CHX and CPC stock solutions in the respective nutrient broth yielding CHX and CPC in concentrations from 128 to 0.25 μg/mL. MICs were examined for CHX and CPC over 10 passages by employing a broth microdilution method, modified from previous works [7,29]. An overnight culture of the respective strain was adjusted to an optical density (OD) of 0.6, as measured with a spectrophotometer at 600 nm (Ultrospec 3300; Amersham Biosciences, Amersham, UK). 200 μL of these bacterial suspensions were added to wells of a 48-well flat-bottom polystyrene microtiter plate (Corning^®^ Costar^®^, Corning, NY, USA) that contained 200 μL of the respective antiseptic in the respective concentrations yielding an end volume of 400 μL in each well. After anaerobic incubation at 37 °C for 24 h, the MICs were determined by visual examination. The well with the highest antiseptic concentration that still exhibited bacterial growth (turbidity) was defined as sub-MIC. The content of this sub-MIC well was added to 5 mL of fresh nutrient broth without antiseptic and incubated at 37 °C overnight. Then, a second passage of MIC evaluation and re-growth was performed as described above. This procedure was performed for 10 passages (P1 to P10) with at least three independent replicates each. Replicates that showed at least 4-fold higher MICs at P10 as compared to P1 were stored at −80 °C for further experiments.

MIC passaging was performed with at least three independent biological replicates per each tested isolate. Furthermore, median (1st; 3rd quartiles) MICs were calculated on a species level using SPSS v. 26 (SPSS Inc., Chicago, IL, USA). For evaluating the stability of the found phenotypic adaptations, the frozen P10 cultures exhibiting at least 4-fold MIC-increase were thawed and cultured in fresh nutrient broth without antiseptic at 37 °C for 3 passages and medium was refreshed every 24 h. MICs were examined on day 1 and day 3, as described above (re-evaluation, R). Isolates showing at least 8-fold MIC-increases and their respective WT strains were further evaluated in terms of phenotypic antibiotic resistance testing, biofilm formation capacity and determination of antibiotic resistance genes.

### 2.4. Biofilm Formation Capacity

The microtiter plate test for biofilm formation was conducted for those P10 replicates exhibiting at least 8-fold MIC increases and their WT strains, as it was described earlier in detail [36,37]. In brief, an overnight culture of each isolate was prepared in tryptic soy broth (TSB; Merck) under aerobic conditions with 5% CO_2_ at 37 °C. The number of colony forming units (CFU) of each overnight culture, as determined on Columbia blood agar, was in the range of 10^8^ CFU/mL. 180 µL fresh TSB were pipetted into each well of polystyrene 96-well tissue culture plates (Greiner Bio-One, Frickenhausen, Germany). Then, 20 µL of the overnight culture were added to each well. The plates were incubated at 37 °C in an aerobic atmosphere with 5% CO_2_ for 24 h. The culture medium was discharged, and the 96-well-plates were washed three times using 300 µL phosphate buffered saline (PBS, Sigma-Aldrich) to remove the non-adherent bacteria. The plates were air-dried at room temperature and the adherent microorganisms were stained with Carbol Gentiana Violet solution (Carl Roth, Karlsruhe, Germany) for 10 min. Afterwards, the wells were rinsed with dH_2_O to remove the excess dye. The plates were then dried for 10 min at 60 °C and 100 µL of absolute ethanol (99.9% *v*/*v*; Merck) were added to each well to solubilize the dye from the stained biofilms. The OD of the solubilized dye was measured using a Tecan Infinite-M200Plate-Reader (Tecan, Crailsheim, Germany) at a wavelength of 595 nm (OD_595_). The strain *Enterococcus faecalis* T9 described by Maekawa et al. [38] was used as positive control for biofilm formation. All experiments were conducted 8-fold and the median (1st; 3rd quartiles) values were determined after subtraction of the OD_595_ blank (TSB only) values using SPSS. Data were analyzed statistically using SPSS by applying non-parametric procedures (Mann-Whitney *U* tests; α = 0.05) for pairwise comparisons between P10 replicates and their corresponding WT strains.

### 2.5. Phenotypic Antibiotic Resistance Testing

To assess phenotypic antibiotic resistance, those P10 replicates that showed at least 8-fold MIC increase and their respective WT strains were tested using the Etest method (Liofilchem^®^ MTSTM; Liofilchem, Roseto degli Abruzzi, Italy), as described earlier [37]. In brief, several colonies from each pure culture were picked and a suspension thereof was prepared and adjusted to McFarland 0.5 (equivalent to approximately 10^8^ CFU/mL). Mueller-Hinton-Blood agar plates (MHB agar plates, for aerobic/facultative anaerobic isolates of the genus *Streptococcus*) were inoculated with this suspension. The inoculation of the agar plates was conducted by using a rota-plater (Retro C80TM bioMérieux, Marcy-l’Etoile, France). Following inoculation, the respective Etest strips were placed on the plates. After incubation the results were interpreted using the breakpoints according to EUCAST (European Committee on Antimicrobial Susceptibility Testing) v. 12.0, 2022 if available and susceptibility was determined as susceptible (S), intermediate (I) or resistant (R). If EUCAST values were not available, MIC values for similar strains were taken from previous reports and used to describe the susceptibility of the respective isolates. The following antibiotics were included to characterize the phenotypic susceptibility of all isolates: penicillin G (PenG), ampicillin/amoxicillin (AMP/AMX), cefuroxime (CXM), meropenem (MEM), tetracycline (TET), tigecycline (TGC), clindamycin (CLI), erythromycin (ERY), moxifloxacin (MXF), and vancomycin (VAN).

### 2.6. Determination of the Presence of Antibiotic Resistance Genes (ARGs) by PCR

The presence of antibiotic resistance genes (ARGs) was determined for P10 replicates showing at least 8-fold MIC increase and their respective WT strains. First, DNA from P10 replicates and WT strains was extracted using the DNeasy blood and tissue kit (Qiagen, Hilden, Germany). As described previously [39], the protocol of the manufacturer was modified by adding 30 µL mutanolysin (1500 U/mL) and 20 µL lysozyme (20 mg/mL; both: Sigma-Aldrich) to 150 µL of lysis buffer and incubating for 1.5 h at 37 °C in order to achieve sufficient cell lysis for Gram-positive bacteria.

Primer pairs for different ARGs described in the literature were purchased from Sigma-Aldrich. The sequences of all used primer pairs and their target genes are depicted in the Supplementary Table S1. The total volume of the PCR amplification mixture was 25 µL. The reaction mixture contained 1× PCR buffer, 0.1–0.5 µM of forward and reverse primers, 200–300 µM deoxyribonucleoside triphosphates mix (dNTPs; Peqlab GmbH, Erlangen, Germany), 2.5 U Taq Polymerase (Qiagen, Hilden, Germany) and 1–2 µL of the bacterial DNA, depending on the respective primer pair. To confirm the presence of bacterial DNA, the universal primers 27f-YM and 1492-Rlong were used for the detection of the 16S rRNA gene in each isolate (Supplementary Table S1). The following positive control strains were used to confirm the corresponding PCR reactions: *Enterococcus faecium* 633 (*tetM*), laboratory strain from sewage (*tetO*), *Streptococcus oralis* FG13-1b (*tetA*, *tetB*), *Enterococcus faecalis* 628 (*tetC*, *tetD*, *tetW*), *E. faecium* 401, 643 (*ermB*), *Staphylococcus aureus* MRSA 4331, *S. aureus* MSSA 2250 (*ermC*), laboratory strain *Eikenella corrodens* FG-15-4a (*ermX*), *Streptococcus pneumoniae* 378 (*MefI*), laboratory strain *Streptococcus intermedius* FG-15-11 (*mefAI*), *Klebsiella pneumoniae* 1230 (*blaTEM-1*; *blaCTX-M-1*), *Enterobacter cloacae* 458 (*ampC*), *Fusobacterium nucleatum* HG-10-2aa, HG10-12a (*blaOXA-85*), *Prevotella nigrescens* HP-04-1aa, HP-02-1a (*cfxA*), *S. pneumoniae* ATCC 49619 (*pbpX2*, *int-II*), *S. pneumoniae* DSM 20566 (*patA*, *patB*), *E. faecium* 633, 643 (*vanA*), *E. faecium* 628, 401 (*vanB*), *Enterococcus gallinarium* 766, 767 (*vanC*), *E. coli* DSM 105182 (*mcr-1*), *E. faecalis* ATCC 29212 (*xis-II*). For *tetA1*, *tetC1*, *tetE1*, *ermA*, *ermF*, *mefAII*, *blaCSP-1*, *vanC2/3*, *vanD*, *vanE*, *aph3* and *lsaC*, no positive control strains were available.

PCR was performed in a PCR cycler (Bio-Rad, Hercules, CA, USA) under different cycling conditions (temperature programs) depending on the annealing temperatures of the different primer pairs used (Supplementary Table S1). The PCR amplification products were analyzed by capillary electrophoresis with the QIAxcel Advanced system (Qiagen, Venlo, The Netherlands) using capillary gel electrophoresis. Each sample was diluted 10-fold in dH_2_O, then automatically loaded into an individual capillary (sample injection voltage 10 kV, sample injection time 5 s) and voltage (420 kV) was applied. Migrating DNA molecules through the capillary were detected and measured as a fluorescent signal. After processing, the data were displayed as an electropherogram or gel image.

## 3. Results

### 3.1. MIC Passaging and Re-Evaluation of Phenotypic Adaptation

Table 2 shows the results for the MIC passaging for all investigated isolates summarized on the species level. Detailed results from MIC passaging for each investigated isolate can be found in the Supplementary Table S2.

Out of the 112 *Streptococcus* isolates passaged in CHX, 24 isolates showed a 4-fold and four isolates an 8-fold MIC increase between P1 and P10. With CPC, six isolates showed a 4-fold MIC increase. Two out of the 19 *Actinomyces* isolates passaged in CHX showed a 4-fold MIC increase between P1 and P10, while no MIC changes could be observed for passaging in CPC. Likewise, the 20 *Rothia* isolates showed no changes in MIC development for CHX or CPC between P1 and P10. The 26 *Veillonella* isolates showed no MIC changes between P1 and P10 when passaged in CHX, but one isolate showed a 4-fold MIC increase after CPC passaging.

As described above, all isolates showing a 4- or more-fold increase were re-evaluated after regrowth in antiseptic-free nutrient broth for 24 or 72 h (R24 h or R72 h, respectively). For the CHX passaged samples, the re-evaluation MIC always remained stable or even increased further compared to P1. The same applied for comparison with P10, but for three exceptions. The re-evaluated CPC samples showed identical MIC values or increased MICs compared to P1, but mostly decreased MICs as compared to P10. More detailed information can be found in the Supplementary Table S2.

Table 3 shows details of the isolates that showed 8-fold MIC increase between P1 and P10, namely the streptococcal strains 59, 73, 78 and 93 passaged in CHX. Three out of six replicates of *S. mutans* (strains 59a, 59d, 59f) increased their MICs from 0.5 µg/mL at P1 to 4 µg/mL at P10 and maintained the increased MIC at the R24 h. Strains 59a and 59f showed decreased MICs after R72 h resulting in a 4-fold increase compared to P1 and a halving compared to P10. Replicate 59c showed a stable MIC of 4 µg/mL also at R72 h resulting in an 8-fold increase compared to P1. One out of six replicates of *S. salivarius* (strain 73a) increased its MIC from 1 µg/mL at P1 to 8 µg/mL at P10 and showed a further increased MIC of 16 µg/mL at R24 h, decreasing again to 8 µg/mL at R72 h. This means an 8-fold increase compared to P1 and a stable MIC compared to P10. One out of six replicates of *S. vestibularis* (strain 78e) increased its MIC from 1 µg/mL at P1 to 8 µg/mL at P10, and further to 16 µg/mL at R24 h. The MIC at R72 h decreased to 8 µg/mL resulting in an 8-fold increase compared to P1 and a stable MIC compared to P10. Four out of six replicates of S. mitis (strains 93b, 93c, 93d, 93e) increased their MICs from 1 µg/mL at P1 to 8 µg/mL at P10. After R24 h and R72 h, replicate 93b decreased its MIC to 4 µg/mL and replicates 93c, 93d and 93e showed a stable MIC of 8 µg/mL.

### 3.2. Biofilm Formation Capacity

**Figure 1:**
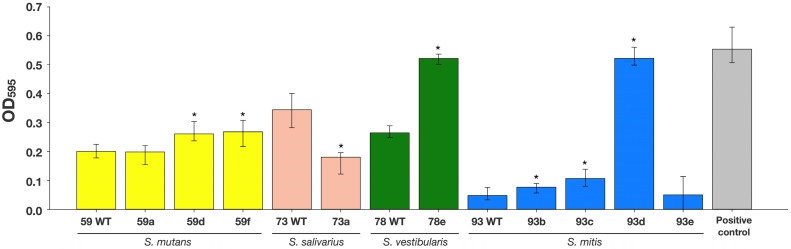
Biofilm formation capacity of P10 replicates showing 8-fold MIC increase toward CHX and corresponding WT strains. Asterisks indicate statistically significant differences from pairwise comparisons between P10 replicates and their respective WT strains. *E. faecalis* strain T9 was used as positive control for biofilm formation capacity [38].

Those P10 replicates of *S. mutans* (strains 59a, 59d, 59f), *S. salivarius* (strain 73a), *S. vestibularis* (strain 78e) and *S. mitis* (strains 93b, 93c, 93d, 93e), which showed at least 8-fold higher MICs after passaging with subinhibitory CHX-concentrations were tested for their biofilm formation capacity as compared to their WT strains. The increase in OD_595_ value, representing an increased percentage of adhered bacterial cells, was the measure for the increase in biofilm formation. The medians of biofilm formation capacity values (measured OD_595_) are shown in Figure 1. Two *S. mutans* P10 replicates (strains 59d and 59f) revealed significantly increased median OD_595_ values of 0.26 and 0.27, respectively, as compared to their WT strain 59 (0.20). The P10 replicate *S. vestibularis* strain 78e also showed significantly biofilm formation capacity (0.52) as compared to the WT strain 78 (0.26). The biofilm formation capacity of three P10 replicates of *S. mitis* (strains 93b, 93c and 93d) was also increased from a median OD_595_ of 0.05 (WT strain 93) to median OD_595_ values from 0.08 to 0.52. Conversely, the P10 replicate *S. salivarius* strain 73a showed a significantly reduced biofilm formation (0.18) as compared to its WT strain 73 (0.34). The biofilm formation capacity of two P10 replicate strains (*S. mutans* 59a, *S. mitis* 93e) was not significantly affected by passaging in subinhibitory CHX-concentrations. *E. faecalis* T9, which was used as positive control for biofilm formation capacity, showed the highest median OD_595_ (0.52) in comparison to all other tested strains.

### 3.3. Phenotypic Antibiotic Resistance

Table 4 depicts the results from phenotypic antibiotic resistance evaluation of the P10 replicates that showed at least 8-fold higher MICs after passaging with subinhibitory CHX-concentrations and their respective WT strains. *S. mutans* strain 59 and its P10 replicates were found susceptible to all tested antibiotics. *S. salivarius* strain 73 and *S. vestibularis* strain 78 and their P10 replicates 73a and 78e were found resistant to ERY (MICs: 73 WT: 8–12 µg/mL; 73a: 4–6 µg/mL; 78 WT: 6 µg/mL; 78e: 3 µg/mL). Strains 73 and 73a also showed intermediate resistance to PenG (MICs: 73 WT: 0.5 µg/mL; 73a: 0.5–0.75 µg/mL) and AMP/AMX (MICs: 73 WT: 0.38–0.75 µg/mL; 73a: 0.5–1.0 µg/mL). Apart from that, strains 73, 73a, 78 and 78e were susceptible to all other tested antibiotics. *S. mitis* strain 93 and its P10 replicates 93b, 93c, 93d and 93e were found resistant to TET (MICs: 93 WT: 12–24 µg/mL; 93b: 16–24 µg/mL; 93c: 12–24 µg/mL; 93d: 12 µg/mL; 93e: 12–24 µg/mL) and ERY (MICs: 93 WT: 6–8 µg/mL; 93b: 8–24 µg/mL; 93c: 8–24 µg/mL; 93d: 2 µg/mL; 93e: 12–24 µg/mL). Furthermore, strains 93b, 93d and 93e showed some intermediate or resistant values toward AMP/AMX (MICs: 93b: 0.5–0.75 µg/mL; 93d: 0.38–0.5 µg/mL) and CXM (MICs: 93b: 0.75–1 µg/mL; 93e: 0.5–0.75 µg/mL), which, however, were just within one MIC-step as compared with the MICs of the WT strain 93. Likewise, such slight fluctuations within one MIC-step were detected when comparing the MICs of WT strains and their respective P10 replicate strains.

### 3.4. Presence of ARGs

All P10 replicates, which showed at least 8-fold higher MICs at P10 as compared to P1, and their respective WT strains were tested for the presence of 36 different ARGs, as shown in Table 5. The detected genes confer resistance against a diverse array of antibiotics including tetracycline, β-lactams, streptogramines, fluoroquinolones, vancomycin, colistin, erythromycin, cephamycin, aminoglycosides, lincomycin and clindamycin. Additionally, resistance genes for efflux pumps and excision of Tn916 were analysed by PCR.

All isolates gave a positive result with the universal bacterial primer set. PCR products detecting ARGs were obtained for all *S. mitis* strains (93 WT as well as its P10 replicates 93b, 93c, 93d, 93e), as follows: *tetM* (tetracycline), *patA* and *patB* (fluoroquinolones), *MefI* (macrolides), pbpX2 (cephalosporin, cephamycin, penams) and *int* (integrase) as well as *xis* (excisionase). One *S. mitis* P10 replicate (93d) showed a positive PCR result for *patB* and *pbpX2* but the respective WT strain 93 was negative for these 2 ARGs. *MefI* was also detected in all *S. salivarius* (73 WT, 73a) and *S. vestibularis* strains (78 WT, 78e) before and after CHX-passaging.

## 4. Discussion

The World Health Organization considers free sale of antimicrobial products containing low concentrations of the antimicrobial agent to be a key source of the spread of AMR [40]. In dentistry, antiseptics such as CHX or CPC are used in low concentrations in over-the-counter oral care products such as mouthwashes or toothpastes [7,8,16]. The aim of the present study was to investigate the potential phenotypic adaptation to antiseptics and the development of cross-resistance to antibiotics in oral bacteria upon multiple exposure to subinhibitory concentrations of these antiseptics in vitro.

For this purpose, a modified microdilution method was used, as previously described [7] and also used in previous studies to evaluate the adaptation of bacteria to antiseptics upon repeated exposure to subinhibitory concentrations [29,30,31]. However, these studies used typical laboratory strains of oral bacteria. Since such laboratory strains may have lost some important pathophysiological properties due to multiple sub-culturing in vitro [32], clinical isolates obtained from healthy or caries-active patients were studied in the present work. In particular, early colonizers of dental plaque such as *Streptococcus*, *Actinomyces*, *Rothia* and *Veillonella* spp. were selected because these taxa constitute a large part of the oral microbiota of healthy individuals [41] and play an important role in the early stages of oral biofilm formation [42] and biofilm matrix production [43]. Moreover, the first reports of streptococcal adaptation were published as early as the 1970s, shortly after the introduction of CHX into dental practice [26,27,28].

The MICs determined in P1 of the 10-day MIC passaging for the streptococcal isolates were generally in higher concentration ranges than described in the literature for laboratory strains [44,45,46,47,48]. For instance, McBain et al. reported a MIC of 3.9 µg/mL for a laboratory strain of *S. sanguinis* [47], whereas we found a median MIC of 12 µg/mL for the 18 clinical *S. sanguinis* isolates. Similarly, Kaspar et al. found MICs of 1.5 µg/mL for a laboratory strain of *S. mutans*, whereas we found a median MIC of 4 µg/mL for the 18 clinical *S. mutans* isolates [48]. This may be due to the fact that clinical oral isolates are likely to have already been exposed to CHX due to its widespread use in dentistry, which may have resulted in some low-level adaptation to this antiseptic [8]. So Yeon and Si Young also examined clinical streptococcal isolates and reported lower MICs than in the present study. For example, they found a mean MIC of 1.95 µg/mL for their ten *S. anginosus* isolates, whereas our eleven *S. anginosus* isolates exhibited a median MIC of 8 µg/mL. Likewise, our clinical *Streptococcus* strains also showed higher median MICs for CPC as compared to the mean MICs reported by So Yeon and Si Young [46]. In contrast, other taxa had lower MICs in the present study than reported for laboratory strains in other studies. For example, the median MIC for the seven *A. naeslundii* isolates in our study was 1 µg/mL, whereas McBain et al. reported a MIC of 1.95 µg/mL for a laboratory strain [47].

After ten passages in subinhibitory concentrations using a similar microdilution method as in our study, Verspecht et al. observed a mean 1.8-fold MIC increase for CHX for *S. mutans* and a mean 3.15-fold increase for *S. sobrinus*. With respect to CPC, *S. mutans* showed a 1.76-fold increase in MIC and *S. sobrinus* even a nearly 6-fold increase [31]. Kitagawa et al. did not observe an increase in MICs for CHX or CPC for *S. mutans* UA159 [30]. In a previous study by our group, we found 2-fold MIC increases for laboratory strains of *S. mutans* and *A. naeslundii* after MIC-passaging, but these were not stable after culture in antiseptic-free nutrient broth [29]. While there are no clear frameworks for defining resistance to antiseptics [8,49], an increase in MIC by a factor of at least four upon repeated exposure can be considered clinically relevant [49]. If a suchlike adaptation is stable following culture in antiseptic-free nutrient broth, it can be defined as “decreased susceptibility” [6] or “resistance” [49]. Here, we found that 27 isolates showed a 4-fold and four an 8-fold MIC increase to CHX, while seven isolates exhibited a 4-fold MIC increase to CPC when comparing MICs at P1 to those at P10. The isolates that exhibited an 8-fold MIC increase to CHX either showed stable MICs, even after culture in antiseptic-free nutrient broth or still exhibited at least a 4-fold MIC increase compared to P1 and, thus, can be considered as “resistant” or with “decreased susceptibility” according to the definitions outlined above [6,49]. *Streptococci* were already brought into focus in the 1970s in the context of a possible decreased susceptibility to CHX after long-term clinical use of CHX-containing mouthwash, toothpaste or gel [26,27,28]. In this context, however, it should be kept in mind that investigating the bacterial adaptation to antiseptics in vitro does not necessarily reflect the use of antiseptics in real life [50]. For example, antiseptics are usually used in formulations that contain various excipients that may enhance their antibacterial activity [50]. Moreover, the concentrations used in clinical applications are many times higher than the subinhibitory concentrations applied for the MIC-passaging in the present study [50]. However, the standard mode of growth of bacteria in a clinical environment is not as pure cultures, but in polymicrobial biofilms [42]. The biofilm matrix thereby acts as a kind of diffusion barrier for positively-charged molecules such as most antiseptics [8,43,51]. Therefore, these low concentrations tested here for the MIC-passaging can still be reached in deep layers of oral biofilms, although the actual concentrations used were much higher [7,8,43].

The development of AMR has been closely linked to the bacterial biofilm-lifestyle [52]. For instance, clinical isolates of *E. faecalis* were recently shown to increase their capacity to form biofilms in the presence of subinhibitory concentrations of antibiotics, particularly fosfomycin, tetracyclines and vancomycin [36]. Subinhibitory concentrations of mupirocin were also found to stimulate biofilm formation in clinical isolates of *Staphylococcus aureus* by up-regulating holin-/antiholin-like proteins encoded by *cidA*, which are known to modulate cell death and lysis during biofilm formation [53]. On the other hand, Gajdàcs et al. studied 302 clinical isolates of multidrug-resistant (MDR) and non-MDR *Pseudomonas aeruginosa* but found no correlation between MDR, biofilm formation, and other virulence factors except pyocyanin production [54]. However, as they used only phenotypic methods, increased expression of virulence factors under selection pressures such as during exposure to subinhibitory antibiotic concentrations cannot be excluded. Accordingly, Nassar et al. described a correlation between virulence traits such as biofilm formation and antibiotic resistance by combining phenotypic, biochemical, and genetic analyses in their study of 113 clinical isolates of *P. aeruginosa* [55]. While *streptococci* are generally known to be strong biofilm producers [56], most P10 replicates exhibited a significantly increased biofilm formation capacity as compared to their WT strains. A possible explanation for this observation could be a stress response leading to a change in gene expression that confers increased formation of extracellular polysaccharides. In a recent RNA-Seq study performed by our group, we could show that sublethal treatment of *S. mutans* with CHX led to up-regulation of pathways such as glycan biosynthesis, which are associated with increased biofilm formation [57]. Therefore, repeated exposure to subinhibitory concentrations of CHX, as in the present study, could lead to a similar transcriptomic stress response, explaining the increased biofilm formation capacity of most P10 strains as compared to their WT strains.

Since multiple exposure to subinhibitory CHX has also been suspected to induce cross-resistances against antibiotics such as nalidixic acid, tobramycin or colistin in pathogenically relevant bacteria such as *Neisseria gonorrhoeae*, *Salmonella* spp., *Klebsiella pneumoniae* [9,10,58], and similar observations could be made with respect to CPC [7], phenotypic antibiotic resistance was evaluated. Three *S. mitis* P10 replicates were found to have slightly elevated MICs to the ß-lactam antibiotics amoxicillin/ampicillin and cefuroxime. Development of resistance to ß-lactam antibiotics was already observed by Doern et al. for viridans *streptococci* and especially *S. mitis* [59]. They examined 352 blood culture isolates and found continuously increasing rates of resistance to ß-lactam antibiotics in viridans streptococci over a 17-year period, which were more pronounced in *S. mitis* compared with *S. milleri*, *S. salivarius* and *S. sanguinis* [59].

Detection of the antibiotic resistance gene *MefI* [60] was positive in the WT strains and P10 replicates of *S. salivarius* (strain 73), *S. vestibularis* (strain 78), and *S. mitis* (strain 93), consistent with their phenotypic antibiotic resistance to erythromycin. In addition, the WT and P10 replicates of *S. mitis* (strain 93) tested positive for *tetM*, *int-II* and *xis-II*, which may belong to the Tn916 transposon carrying the *tetM* gene, as well as an integrase and an excisionase encoding transposition functions [61] that may confer the phenotypic resistance to tetracycline. In addition, the *xis*-encoded excisionase [61] and the *int*-encoded integron system play an important role in resistance development and its spread via horizontal gene transfer by incorporating foreign genetic material as so called gene cassettes [62,63]. The gene *pbpX2* detected in 93d confers resistance to penams such as amoxicillin or ampicillin and cephalosporins such as cefuroxime [64], but strain 93d was found phenotypically susceptible to these antibiotics. Likewise, WT and P10 strains of 93 were not found to be phenotypically resistant to the fluoroquinolone moxifloxacin, although they tested positive for *patA* and 93d also for *patB* [65]. These genes encode an ATP-binding cassette transporter (ABC transporter) consisting of two subunits, PatA and PatB, and are separated by a gene probably encoding for a transposase, whose role has not been fully elucidated [66,67,68]. PatA or PatB are not functional separately but only together, making dysfunction of this ABC transporter due to mutations a possible scenario [66]. Although *pbpX2*, *patA* and *patB* did not confer phenotypic resistance in the present experiments, these genes are nevertheless part of the genomes of the respective strains and could be transferred to other strains via horizontal gene transfer, highlighting the role of the oral microbiome as a potential reservoir for ARGs [11,12,13].

Further insights into mechanisms leading to decreased susceptibility or resistance to antiseptics and potentially concomitant cross-resistance to antibiotics should ideally combine investigations of adapted strains at the genomic and transcriptomic levels, as recently demonstrated by Kim et al. for benzalkoniumchloride (BAC)-resistant *Pseudomonas aeruginosa* [69], a species also known for its intrinsic resistance to CHX [70,71]. In this study, *P. aeruginosa* strains derived from river sediments were grown in a bioreactor fed with subinhibitory concentrations of BAC or without BAC for a period of three years and subsequently analyzed by whole genome sequencing (WGS) and transcriptome sequencing (RNA Seq). While at the genome level, mutual mutations among those strains were found only in one polymyxin resistance gene (*pmrB*), marked changes were found at the transcriptome level in terms of upregulation of efflux pump genes and spermidine synthase genes, as well as decreased expression of porins and reduced growth rate [69].

## 5. Conclusions

This study demonstrates that clinical isolates of early colonizers in dental plaque can phenotypically adapt to antiseptics such as CHX and CPC upon multiple exposures to subinhibitory concentrations. While there was little change in phenotypic antibiotic resistance of CHX-adapted strains, the ability to form biofilms was increased in most CHX-adapted strains. Although these results cannot be readily extrapolated to the clinical situation, they may raise awareness of the potential risks associated with the widespread use of oral antiseptics by dentists, and also as a low-concentration ingredient in over-the-counter oral hygiene products. To better understand the mechanisms underlying phenotypic adaptation to CHX or CPC, future studies should examine the changes at the genomic and transcriptomic levels of the phenotypically adapted strains compared to their wild-type strains.

## Figures and Tables

**Table 1:** Clinical oral isolates included in this study.

Taxon	Number of Isolates
*Streptococcus anginosus*	11
*Streptococcus constellatus*	1
*Streptococcus oralis*	24
*Streptococcus sanguinis*	18
*Streptococcus intermedius*	1
*Streptococcus gordonii*	9
*Streptococcus salivarius*	6
*Streptococcus sobrinus*	1
*Streptococcus vestibularis*	1
*Streptococcus downii*	2
*Streptococcus parasanguinis*	6
*Streptococcus mitis*	11
*Streptococcus mutans*	18
*Streptococcus cristatus*	3
*Actinomyces naeslundii*	7
*Actinomyces oris*	5
*Actinomyces odontolyticus*	7
*Rothia aeria*	7
*Rothia dentocariosa*	6
*Rothia mucilaginosa*	7
*Veilonella atypica*	10
*Veilonella dispar*	2
*Veilonella parvula*	14

**Table 2:** Results of MIC passaging in CHX or CPC summarized on a species-level.

	CHX					CPC				
Strains	MIC [µg/mL] at P1 ^#^	MIC [µg/mL] at P10 ^#^	FC P1–P10	Numbers of Isolates with		MIC [µg/mL] at P1 ^#^	MIC [µg/mL] at P10 ^#^	FC P1–P10	Numbers of Isolates with	
				4-Fold	8-Fold				4-Fold	8-Fold
				MIC Increase					MIC Increase	
*S. anginosus* *n* = 11	8 (6; 8)	16 (16; 16)	2 (2; 4)	**4**	0	2 (2; 4)	2 (2; 2)	1 (0.5; 1)	0	0
*S. constellatus* *n* = 1	4	4	1	0	0	1 (1; 1)	2 (2; 2)	2 (2; 2)	0	0
*S. oralis* *n* = 24	8 (4; 16)	16 (16; 16)	2 (1; 2.5)	**6**	0	2 (2; 2)	2 (2; 2,5)	1 (1; 1.25)	**1**	0
*S. sanguinis* *n* = 18	12 (5; 28)	16 (8; 16)	1 (0.5; 2)	**1**	0	4 (4; 4)	2 (2; 4)	0.5 (0.5; 1.75)	**2**	0
*S. intermedius* *n* = 1	4	8	2	0	0	8	4	0.5	0	0
*S. gordonii* *n* = 9	4 (4; 8)	16 (8; 16)	2 (2; 4)	**4**	0	2 (2; 4)	4 (2; 4)	1 (0.5; 2)	0	0
*S. salivarius* *n* = 6	4 (2.5; 4)	6 (4; 8)	3 (1.25; 4)	**2**	**1**	2 (2; 2)	2 (2; 2)	1 (1; 1)	0	0
*S. sobrinus* *n* = 1	4	4	1	0	0	2	2	1	0	0
*S. vestibularis* *n* = 1	1	8	8	0	**1**	2	1	0.5	0	0
*S. downii* *n* = 2	4 (4; 4) ˟	16 (16; 16) ˟	4 (4; 4) ˟	**2**	0	3 (2; 4) ˟	1 (2; 2) ˟	0.38 (0.25; 0:5) ˟	0	0
*S. parasanguinis* *n* = 6	4 (4; 7)	16 (16; 16)	3 (2; 4)	**3**	0	4 (2.5; 4)	2 (2; 2)	0.5 (0.5; 0.88)	0	0
*S. mitis* *n* = 11	2 (2; 4)	8 (4; 8)	2 (2; 3)	**2**	**1**	1 (1; 1)	2 (2; 2)	2 (1.5; 2)	**1**	0
*S. mutans* *n* = 18	4 (4; 4)	4 (4; 4)	1 (1; 1)	0	**1**	2 (2; 2)	4 (2; 4)	2 (1; 2)	**1**	0
*S. cristatus* *n* = 3	2 (2; 3)	4 (4; 6)	2 (2; 2)	0	**0**	1 (1; 1.5)	4 (2.5; 4)	2 (1.5; 3)	**1**	0
*A. naeslundii* *n* = 7	1 (0.75; 2)	2 (1.5; 2)	2 (1; 2)	0	0	2 (1.5; 2)	2 (2; 2)	1 (1; 1.5)	0	0
*A. oris* *n* = 5	2 (1; 2)	2 (2; 4)	1 (1; 4)	**2**	0	4 (4; 4)	2 (2; 2)	0.5 (0.5; 1)	0	0
*A. odontolyticus* *n* = 7	4 (3; 4)	4 (2; 4)	1 (0.75; 1)	0	0	4 (4; 4)	2 (2; 2)	0.5 (0.5; 0.5)	0	0
*R. aeria* *n* = 7	2 (2; 4)	4 (2; 4)	1 (1; 2)	0	0	2 (2; 4)	2 (2; 2)	1 (0.5; 1)	0	0
*R. dentocariosa* *n* = 6	4 (4; 4)	4 (4; 4)	1 (1; 1)	0	0	2 (2; 2)	2 (2; 2)	1 (1; 1)	0	0
*R. mucilaginosa* *n* = 7	4 (3; 4)	4 (4; 6)	2 (1; 2)	0	0	2 (1; 2)	2 (2; 2)	1 (1; 1.5)	0	0
*V. atypica* *n* = 10	1 (1; 1)	2 (1; 2)	2 (1; 2)	0	0	1 (1; 1.75)	2 (1.25; 2)	2 (1; 2)	0	0
*V. dispar* *n* = 2	2 (2; 2) ˟	1 (1; 1) ˟	0.5 (0.5; 0.5) ˟	0	0	0.75 (0.625; 0.875) ˟	2 (2; 2) ˟	3 (2.5; 3.5) ˟	**1**	0
*V. parvula* *n* = 14	2 (1; 2)	1.5 (1; 2)	1 (1; 1)	0	0	1.5 (1; 2)	2 (2; 2)	2 (1; 2)	0	0

^#^ MICs are shown on a species level as medians (1st and 3rd quartiles; for *n* ≥ 3), medians (minimum and maximum; for *n* = 2; marked by ˟) or single values (for *n* = 1). P1: passage 1; P10: passage 10; FC: fold-change.

**Table 3:** Isolates exhibiting at least 8-fold MIC increase toward CHX between P1 and P10.

Strain		MIC_CHX_ (µg/mL)				FC P1–P10	FC P1–R72 h	FC P10–R72 h
		P1	P10	R24 h	R72 h			
*S. mutans*	59a	0.5	4	4	2	8	4	0.5
	59b	0.5	0.5	*	*	1	*	*
	59c	0.5	2	*	*	4	*	*
	59d	0.5	4	4	4	8	8	1
	59e	-	-	-	-	-	-	-
	59f	0.5	4	4	2	8	4	0.5
*S. salivarius*	73a	1	8	16	8	8	8	1
	73b	1	2	*	*	2	*	*
	73c	1	4	*	*	4	*	*
	73d	1	4	*	*	4	*	*
	73e	1	4	*	*	4	*	*
	73f	1	4	*	*	4	*	*
*S. vestibularis*	78a	1	4	*	*	4	*	*
	78b	1	4	*	*	4	*	*
	78c	2	4	*	*	2	*	*
	78d	2	8	*	*	4	*	*
	78e	1	8	16	8	8	8	1
	78f	2	4	*	*	2	*	*
*S. mitis*	93a	1	4	*	*	4	*	*
	93b	1	8	4	4	8	4	0.5
	93c	1	8	8	8	8	8	1
	93d	1	8	8	8	8	8	1
	93e	1	8	8	8	8	8	1
	93f	1	4	*	*	4	*	*

MICs of all replicates of the six isolates with at least 8-fold MIC-increase are shown at P1, P10, R24 h, and R72 h. Grey font depicts replicates not reaching an 8-fold MIC increase toward CHX from P1 to P10. P1: passage 1; P10: passage 10; R24 h: re-evaluation after culture in antiseptic-free nutrient broth for 24 h; R72 h: re-evaluation after culture in antiseptic-free nutrient broth for 72 h; FC: fold-change.

**Table 4:** Phenotypic antibiotic resistance of P10 replicates showing 8-fold MIC increase toward CHX and corresponding WT strains.

		*S. mutans*				*S. salivarius*		*S. vestibularis*		*S. mitis*				
		59 WT	59a	59d	59f	73 WT	73a	78 WT	78e	93 WT	93b	93c	93d	93e
β-lactams	PenG	0.023 S	0.023 S	0.023 S	0.023 S	0.5 I	0.5–0.75 I	0.064 S	0.094 S	0.19 S	0.25 S	0.125 S	0.016–0.25 S	0.19 S
	AMP /AMX	0.047 S	0.047 S	0.047 S	0.047 S	0.38–0.75 S/I	0.5–1.0 S/I	0.064 S	0.064 S	0.38 S	0.5–0.75 S/I	0.38 S	0.38–0.5 I	0.38 S
	CXM	0.032 S	0.032 S	0.047 S	0.032 S	0.125–0.19 S	0.19 S	0.047 S	0.032 S	0.5 S	0.75–1 **R**	0.5 S	0.032–0.5 S	0.5–0.75 S/**R**
	MEM	0.064 S	0.064 S	0.064 S	0.064 S	0.19 S	0.25–0.38 S	0.032 S	0.047 S	0.19–0.25 S	0.38 S	0.25–0.38 S	0.032–0.25 S	0.25–0.38 S
Tetracyclines	TET	0.19 S	0.19 S	0.19 S	0.19 S	0.19 S	0.19–0.25 S	0.19 S	0.19 S	12–24 **R**	16–24 **R**	12–24 **R**	12 **R**	12–24 **R**
	TGC	0.064 S	0.064 S	0.064 S	0.064 S	0.047–0.064 S	0.047–0.064 S	0.032 S	0.047 S	0.047–0.064 S	0.047–0.064 S	0.016–0.064 S	0.016–0.047 S	0.032–0.047 S
Lincosamide	CLI	0.064 S	0.064 S	0.064 S	0.064 S	0.047 S	0.047 S	0.032 S	0.047 S	0.094 S	0.094 S	0.047–0.094 S	0.094 S	0.094 S
Macrolide	ERY	0.047 S	0.047 S	0.047 S	0.047 S	8–12 **R**	4–6 **R**	6 **R**	3 **R**	6–8 **R**	8–24 **R**	8–24 **R**	2 **R**	12–24 **R**
Fluoro-quinolone	MXF	0.25 S	0.38 S	0.25 S	0.25 S	0.125–0.19 S	0.125–0.19 S	0.19 S	0.19 S	0.19–0.25 S	0.19 S	0.064–0.094 S	0.125–0.19 S	0.094–0.125 S
Glycopeptide	VAN	0.75 S	0.5 S	0.5 S	0.75 S	0.5 S	0.5–0.75 S	0.5 S	0.75 S	0.25 S	0.38 S	0.25–0.38 S	0.38 S	0.5–0.38 S

The first line shows the respective Etest result, while the second line gives the interpretation according to EUCAST 12.0 (S: susceptible; I: intermediate; **R**: resistant).

**Table 5:** Antibiotic resistance genes (ARGs) of P10 replicates showing 8-fold MIC increase toward CHX and corresponding WT strains as detected by PCR.

ARG	*S. mutans*				*S. salivarius*		*S. vestibularis*		*S. mitis*					Negative Control
	59 WT	59a	59d	59f	73 WT	73a	78 WT	78e	93 WT	93b	93c	93d	93e	
*tetM*	−	−	−	−	−	−	−	−	**+**	**+**	**+**	**+**	**+**	−
tetO	−	−	−	−	−	−	−	−	−	−	−	−	−	−
*tetW*	−	−	−	−	−	−	−	−	−	−	−	−	−	−
*tetA-1*	−	−	−	−	−	−	−	−	−	−	−	−	−	−
*tetB-1*	−	−	−	−	−	−	−	−	−	−	−	−	−	−
*tetC-1*	−	−	−	−	−	−	−	−	−	−	−	−	−	−
*tetD-1*	−	−	−	−	−	−	−	−	−	−	−	−	−	−
*tetE-1*	−	−	−	−	−	−	−	−	−	−	−	−	−	−
*bla*_TEM1_	−	−	−	−	−	−	−	−	−	−	−	−	−	−
*cfxA*	−	−	−	−	−	−	−	−	−	−	−	−	−	−
*bla*_CTX-M-1_	−	−	−	−	−	−	−	−	−	−	−	−	−	−
*bla*_CSP-1_	−	−	−	−	−	−	−	−	−	−	−	−	−	−
*bla*_OXA-85_	−	−	−	−	−	−	−	−	−	−	−	−	−	−
*ampC*	−	−	−	−	−	−	−	−	−	−	−	−	−	−
*pbpX2*	−	−	−	−	−	−	−	−	−	−	−	**+**	−	−
*ermA*	−	−	−	−	−	−	−	−	−	−	−	−	−	−
*ermB*	−	−	−	−	−	−	−	−	−	−	−	−	−	−
*ermC*	−	−	−	−	−	−	−	−	−	−	−	−	−	−
*ermF*	−	−	−	−	−	−	−	−	−	−	−	−	−	−
*MefI*	−	−	−	−	**+**	**+**	**+**	**+**	**+**	**+**	**+**	**+**	**+**	−
*mefAI*	−	−	−	−	−	−	−	−	−	−	−	−	−	−
*mef A II*	−	−	−	−	−	−	−	−	−	−	−	−	−	−
*patA*	−	−	−	−	−	−	−	−	**+**	**+**	**+**	**+**	**+**	−
*patB*	−	−	−	−	−	−	−	−	−	−	−	**+**	−	−
*vanA*	−	−	−	−	−	−	−	−	−	−	−	−	−	−
*vanB*	−	−	−	−	−	−	−	−	−	−	−	−	−	−
*vanC1*	−	−	−	−	−	−	−	−	−	−	−	−	−	−
*vanC2/3*	−	−	−	−	−	−	−	−	−	−	−	−	−	−
*vanD*	−	−	−	−	−	−	−	−	−	−	−	−	−	−
*vanE*	−	−	−	−	−	−	−	−	−	−	−	−	−	−
*mcr-1*	−	−	−	−	−	−	−	−	−	−	−	−	−	−
*lsaC*	−	−	−	−	−	−	−	−	−	−	−	−	−	−
*aph3*	−	−	−	−	−	−	−	−	−	−	−	−	−	−
*int-II*	−	−	−	−	−	−	−	−	**+**	**+**	**+**	**+**	**+**	−
*xis-II*	−	−	−	−	−	−	−	−	**+**	**+**	**+**	**+**	**+**	−
positive control	**+**	**+**	**+**	**+**	**+**	**+**	**+**	**+**	**+**	**+**	**+**	**+**	**+**	−

Corresponding primers are depicted in Supplementary Table S1. −: negative PCR result, +: positive PCR result.

## Data Availability

The datasets used or analyzed during this study are available from the corresponding author on reasonable request.

## Supplementary Materials

The following supporting information can be downloaded at: https://www.mdpi.com/article/10.3390/antibiotics11050688/s1, Supplementary Table S1: Primers used for the detection of antibiotic resistance genes (ARGs); Supplementary Table S2: Detailed results from MIC passaging for all clinical oral isolates included in this study. References [60,61,65,72,73,74,75,76,77,78,79,80,81,82,83,84,85,86,87,88,89,90,91,92,93] are cited in the supplementary materials.

## References

[1] Tacconelli E., Pezzani M.D. (2019). Public health burden of antimicrobial resistance in Europe. Lancet Infect. Dis..

[2] O’Neill J. (2016). Tackling Drug-Resistant Infections Globally: Final Report and Recommendations.

[3] Murray C.J.L., Ikuta K.S., Sharara F., Swetschinski L., Robles Aguilar G., Gray A., Han C., Bisignano C., Rao P., Wool E. (2022). Global burden of bacterial antimicrobial resistance in 2019: A systematic analysis. Lancet.

[4] Laxminarayan R. (2022). The overlooked pandemic of antimicrobial resistance. Lancet.

[5] Lu J., Guo J. (2021). Disinfection spreads antimicrobial resistance. Science.

[6] Merchel Piovesan Pereira B., Tagkopoulos I. (2019). Benzalkonium Chlorides: Uses, Regulatory Status, and Microbial Resistance. Appl. Environ. Microbiol..

[7] Mao X., Auer D.L., Buchalla W., Hiller K.-A., Maisch T., Hellwig E., Al-Ahmad A., Cieplik F. (2020). Cetylpyridinium Chloride: Mechanism of Action, Antimicrobial Efficacy in Biofilms, and Potential Risks of Resistance. Antimicrob. Agents Chemother..

[8] Cieplik F., Jakubovics N.S., Buchalla W., Maisch T., Hellwig E., Al-Ahmad A. (2019). Resistance Toward Chlorhexidine in Oral Bacteria—Is There Cause for Concern?. Front. Microbiol..

[9] Wand M.E., Bock L.J., Bonney L.C., Sutton J.M. (2017). Mechanisms of Increased Resistance to Chlorhexidine and Cross-Resistance to Colistin following Exposure of Klebsiella pneumoniae Clinical Isolates to Chlorhexidine. Antimicrob. Agents Chemother..

[10] Laumen J.G.E., van Dijck C., Manoharan-Basil S.S., Abdellati S., de Baetselier I., Cuylaerts V., de Block T., van den Bossche D., Xavier B.B., Malhotra-Kumar S. (2021). Sub-Inhibitory Concentrations of Chlorhexidine Induce Resistance to Chlorhexidine and Decrease Antibiotic Susceptibility in Neisseria gonorrhoeae. Front. Microbiol..

[11] Roberts A.P., Mullany P. (2010). Oral biofilms: A reservoir of transferable, bacterial, antimicrobial resistance. Expert Rev. Anti-Infect. Ther..

[12] Jiang S., Zeng J., Zhou X., Li Y. (2018). Drug Resistance and Gene Transfer Mechanisms in Respiratory/Oral Bacteria. J. Dent. Res..

[13] Arredondo A., Blanc V., Mor C., Nart J., León R. (2020). Tetracycline and multidrug resistance in the oral microbiota: Differences between healthy subjects and patients with periodontitis in Spain. J. Oral Microbiol..

[14] Haps S., Slot D.E., Berchier C.E., van der Weijden G.A. (2008). The effect of cetylpyridinium chloride-containing mouth rinses as adjuncts to toothbrushing on plaque and parameters of gingival inflammation: A systematic review. Int. J. Dent. Hyg..

[15] Figuero E., Herrera D., Tobías A., Serrano J., Roldán S., Escribano M., Martín C. (2019). Efficacy of adjunctive anti-plaque chemical agents in managing gingivitis: A systematic review and network meta-analyses. J. Clin. Periodontol..

[16] Sanz M., Serrano J., Iniesta M., Santa Cruz I., Herrera D. (2013). Antiplaque and antigingivitis toothpastes. Monogr. Oral Sci..

[17] Cieplik F., Kara E., Muehler D., Enax J., Hiller K.-A., Maisch T., Buchalla W. (2019). Antimicrobial efficacy of alternative compounds for use in oral care toward biofilms from caries-associated bacteria in vitro. Microbiologyopen.

[18] Mohd-Said S., Mohd-Dom T.N., Suhaimi N., Rani H., McGrath C. (2021). Effectiveness of Pre-procedural Mouth Rinses in Reducing Aerosol Contamination During Periodontal Prophylaxis: A Systematic Review. Front. Med..

[19] Gottsauner M.J., Michaelides I., Schmidt B., Scholz K.J., Buchalla W., Widbiller M., Hitzenbichler F., Ettl T., Reichert T.E., Bohr C. (2020). A prospective clinical pilot study on the effects of a hydrogen peroxide mouthrinse on the intraoral viral load of SARS-CoV-2. Clin. Oral Investig..

[20] Meister T.L., Gottsauner J.-M., Schmidt B., Heinen N., Todt D., Audebert F., Buder F., Lang H., Gessner A., Steinmann E. (2022). Mouthrinses against SARS-CoV-2—high antiviral effectivity by membrane disruption in vitro translates to mild effects in a randomized placebo-controlled clinical trial. Virus Res..

[21] Waldron C., Nunn J., Mac Giolla Phadraig C., Comiskey C., Guerin S., Harten M.T., Donnelly-Swift E., Clarke M.J. (2019). Oral hygiene interventions for people with intellectual disabilities. Cochrane Database Syst. Rev..

[22] Pithon M.M., Sant’Anna L.I.D.A., Baião F.C.S., Santos R.L.d., Da Coqueiro R.S., Maia L.C. (2015). Assessment of the effectiveness of mouthwashes in reducing cariogenic biofilm in orthodontic patients: A systematic review. J. Dent..

[23] Solderer A., Kaufmann M., Hofer D., Wiedemeier D., Attin T., Schmidlin P.R. (2019). Efficacy of chlorhexidine rinses after periodontal or implant surgery: A systematic review. Clin. Oral Investig..

[24] Grönbeck Lindén I., Hägglin C., van Gahnberg L., Andersson P. (2017). Factors Affecting Older Persons’ Ability to Manage Oral Hygiene: A Qualitative Study. JDR Clin. Transl. Res..

[25] Zhao T., Wu X., Zhang Q., Li C., Worthington H.V., Hua F. (2020). Oral hygiene care for critically ill patients to prevent ventilator-associated pneumonia. Cochrane Database Syst. Rev..

[26] Emilson C.G., Ericson T., Heyden G., Lilia J. (1972). Effect of chlorhexidine on human oral streptococci. J. Periodont. Res..

[27] Schiott C.R., Löe H. (1972). The sensitivity of oral streptococci to chlorhexidine. J. Periodont. Res..

[28] Emilson C.G., Fornell J. (1976). Effect of toothbrushing with chlorhexidine gel on salivary microflora, oral hygiene, and caries. Eur. J. Oral Sci..

[29] Schwarz S.R., Hirsch S., Hiergeist A., Kirschneck C., Muehler D., Hiller K.-A., Maisch T., Al-Ahmad A., Gessner A., Buchalla W. (2021). Limited antimicrobial efficacy of oral care antiseptics in microcosm biofilms and phenotypic adaptation of bacteria upon repeated exposure. Clin. Oral Investig..

[30] Kitagawa H., Izutani N., Kitagawa R., Maezono H., Yamaguchi M., Imazato S. (2016). Evolution of resistance to cationic biocides in Streptococcus mutans and Enterococcus faecalis. J. Dent..

[31] Verspecht T., Rodriguez Herrero E., Khodaparast L., Khodaparast L., Boon N., Bernaerts K., Quirynen M., Teughels W. (2019). Development of antiseptic adaptation and cross-adapatation in selected oral pathogens in vitro. Sci. Rep..

[32] Fux C.A., Shirtliff M., Stoodley P., Costerton J.W. (2005). Can laboratory reference strains mirror “real-world” pathogenesis?. Trends Microbiol..

[33] Al-Ahmad A., Auschill T.M., Braun G., Hellwig E., Arweiler N.B. (2006). Overestimation of Streptococcus mutans prevalence by nested PCR detection of the 16S rRNA gene. J. Med. Microbiol..

[34] Bernardi S., Karygianni L., Filippi A., Anderson A.C., Zürcher A., Hellwig E., Vach K., Macchiarelli G., Al-Ahmad A. (2020). Combining culture and culture-independent methods reveals new microbial composition of halitosis patients’ tongue biofilm. Microbiologyopen.

[35] Cieplik F., Wiedenhofer A.M., Pietsch V., Hiller K.-A., Hiergeist A., Wagner A., Baldaranov D., Linker R.A., Jantsch J., Buchalla W. (2020). Oral Health, Oral Microbiota, and Incidence of Stroke-Associated Pneumonia-A Prospective Observational Study. Front. Neurol..

[36] Bernardi S., Anderson A., Macchiarelli G., Hellwig E., Cieplik F., Vach K., Al-Ahmad A. (2021). Subinhibitory Antibiotic Concentrations Enhance Biofilm Formation of Clinical Enterococcus faecalis Isolates. Antibiotics.

[37] Al-Ahmad A., Ameen H., Pelz K., Karygianni L., Wittmer A., Anderson A.C., Spitzmüller B., Hellwig E. (2014). Antibiotic Resistance and Capacity for Biofilm Formation of Different Bacteria Isolated from Endodontic Infections Associated with Root-filled Teeth. J. Endod..

[38] Maekawa S., Yoshioka M., Kumamoto Y. (1992). Proposal of a new scheme for the serological typing of Enterococcus faecalis strains. Microbiol. Immunol..

[39] Anderson A.C., Rothballer M., Altenburger M.J., Woelber J.P., Karygianni L., Vach K., Hellwig E., Al-Ahmad A. (2020). Long-Term Fluctuation of Oral Biofilm Microbiota following Different Dietary Phases. Appl. Environ. Microbiol..

[40] World Health Organization (2015). Antimicrobial Resistance Global Action Plan.

[41] Diaz P.I., Hoare A., Hong B.-Y. (2016). Subgingival Microbiome Shifts and Community Dynamics in Periodontal Diseases. J. Calif. Dent. Assoc..

[42] Kolenbrander P.E., Palmer R.J., Periasamy S., Jakubovics N.S. (2010). Oral multispecies biofilm development and the key role of cell-cell distance. Nat. Rev. Microbiol..

[43] Jakubovics N.S., Goodman S.D., Mashburn-Warren L., Stafford G.P., Cieplik F. (2021). The dental plaque biofilm matrix. Periodontol. 2000.

[44] Järvinen H., Pienihäkkinen K., Huovinen P., Tenovuo J. (1995). Susceptibility of Streptococcus mutans and Streptococcus sobrinus to antimicrobial agents after short-term oral chlorhexidine treatments. Eur. J. Oral Sci..

[45] Järvinen H., Tenovuo J., Huovinen P. (1993). In vitro susceptibility of Streptococcus mutans to chlorhexidine and six other antimicrobial agents. Antimicrob. Agents Chemother..

[46] So Yeon L., Si Young L. (2019). Susceptibility of Oral Streptococci to Chlorhexidine and Cetylpyridinium Chloride. Biocontrol Sci..

[47] McBain A.J., Bartolo R.G., Catrenich C.E., Charbonneau D., Ledder R.G., Gilbert P. (2003). Effects of a chlorhexidine gluconate-containing mouthwash on the vitality and antimicrobial susceptibility of in vitro oral bacterial ecosystems. Appl. Environ. Microbiol..

[48] Kaspar J.R., Godwin M.J., Velsko I.M., Richards V.P., Burne R.A. (2019). Spontaneously Arising Streptococcus mutans Variants with Reduced Susceptibility to Chlorhexidine Display Genetic Defects and Diminished Fitness. Antimicrob. Agents Chemother..

[49] Chapman J.S. (2003). Biocide resistance mechanisms. Int. Biodeterior. Biodegrad..

[50] Fox L.J., Kelly P.P., Humphreys G.J., Waigh T.A., Lu J.R., McBain A.J. (2022). Assessing the risk of resistance to cationic biocides incorporating realism-based and biophysical approaches. J. Ind. Microbiol. Biotechnol..

[51] Stewart P.S. (2003). Diffusion in biofilms. J. Bacteriol..

[52] Ciofu O., Moser C., Jensen P.Ø., Høiby N. (2022). Tolerance and resistance of microbial biofilms. Nat. Rev. Microbiol..

[53] Jin Y., Guo Y., Zhan Q., Shang Y., Qu D., Yu F. (2020). Subinhibitory Concentrations of Mupirocin Stimulate Staphylococcus aureus Biofilm Formation by Upregulating *cidA*. Antimicrob. Agents Chemother..

[54] Gajdács M., Baráth Z., Kárpáti K., Szabó D., Usai D., Zanetti S., Donadu M.G. (2021). No Correlation between Biofilm Formation, Virulence Factors, and Antibiotic Resistance in Pseudomonas aeruginosa: Results from a Laboratory-Based In Vitro Study. Antibiotics.

[55] Nassar O., Desouky S.E., El-Sherbiny G.M., Abu-Elghait M. (2022). Correlation between phenotypic virulence traits and antibiotic resistance in Pseudomonas aeruginosa clinical isolates. Microb. Pathog..

[56] Bowen W.H., Burne R.A., Wu H., Koo H. (2018). Oral Biofilms: Pathogens, Matrix, and Polymicrobial Interactions in Microenvironments. Trends Microbiol..

[57] Muehler D., Mao X., Czemmel S., Geißert J., Engesser C., Hiller K.-A., Widbiller M., Maisch T., Buchalla W., Al-Ahmad A. (2022). Transcriptomic Stress Response in Streptococcus mutans following Treatment with a Sublethal Concentration of Chlorhexidine Digluconate. Microorganisms.

[58] Kampf G. (2018). Biocidal Agents Used for Disinfection Can Enhance Antibiotic Resistance in Gram-Negative Species. Antibiotics.

[59] Doern G.V., Ferraro M.J., Brueggemann A.B., Ruoff K.L. (1996). Emergence of high rates of antimicrobial resistance among viridans group streptococci in the United States. Antimicrob. Agents Chemother..

[60] Reinert R.R., Filimonova O.Y., Al-Lahham A., Grudinina S.A., Ilina E.N., Weigel L.M., Sidorenko S.V. (2008). Mechanisms of Macrolide Resistance among Streptococcus pneumoniae Isolates from Russia. Antimicrob. Agents Chemother..

[61] Calatayud L., Ardanuy C., Cercenado E., Fenoll A., Bouza E., Pallares R., Martín R., Liñares J. (2007). Serotypes, Clones, and Mechanisms of Resistance of Erythromycin-Resistant Streptococcus pneumoniae Isolates Collected in Spain. Antimicrob. Agents Chemother..

[62] Partridge S.R., Recchia G.D., Scaramuzzi C., Collis C.M., Stokes H.W., Hall R.M. (2000). Definition of the attI1 site of class 1 integrons. Microbiology.

[63] Collis C.M., Kim M.-J., Partridge S.R., Stokes H.W., Hall R.M. (2002). Characterization of the class 3 integron and the site-specific recombination system it determines. J. Bacteriol..

[64] Nakayama A., Takao A. (2003). β-Lactam resistance in Streptococcus mitis isolated from saliva of healthy subjects. J. Infect. Chemother..

[65] Garvey M.I., Baylay A.J., Wong R.L., Piddock L.J.V. (2011). Overexpression of patA and patB, which encode ABC transporters, is associated with fluoroquinolone resistance in clinical isolates of Streptococcus pneumoniae. Antimicrob. Agents Chemother..

[66] Boncoeur E., Durmort C., Bernay B., Ebel C., Di Guilmi A.M., Croizé J., Vernet T., Jault J.-M. (2012). PatA and PatB form a functional heterodimeric ABC multidrug efflux transporter responsible for the resistance of Streptococcus pneumoniae to fluoroquinolones. Biochemistry.

[67] Feng J., Lupien A., Gingras H., Wasserscheid J., Dewar K., Légaré D., Ouellette M. (2009). Genome sequencing of linezolid-resistant Streptococcus pneumoniae mutants reveals novel mechanisms of resistance. Genome Res..

[68] Garvey M.I., Piddock L.J.V. (2008). The efflux pump inhibitor reserpine selects multidrug-resistant Streptococcus pneumoniae strains that overexpress the ABC transporters PatA and PatB. Antimicrob. Agents Chemother..

[69] Kim M., Hatt J.K., Weigand M.R., Krishnan R., Pavlostathis S.G., Konstantinidis K.T. (2018). Genomic and Transcriptomic Insights into How Bacteria Withstand High Concentrations of Benzalkonium Chloride Biocides. Appl. Environ. Microbiol..

[70] Thomas L., Maillard J.Y., Lambert R.J., Russell A.D. (2000). Development of resistance to chlorhexidine diacetate in Pseudomonas aeruginosa and the effect of a “residual” concentration. J. Hosp. Infect..

[71] Rozman U., Pušnik M., Kmetec S., Duh D., Šostar Turk S. (2021). Reduced Susceptibility and Increased Resistance of Bacteria against Disinfectants: A Systematic Review. Microorganisms.

[72] Lins R.X., de Oliveira Andrade A., Hirata Junior R., Wilson M.J., Lewis M.A., Williams D.W., Fidel R.A.S. (2013). Antimicrobial resistance and virulence traits of Enterococcus faecalis from primary endodontic infections. J. Dent..

[73] Poyart C., Jardy L., Quesne G., Berche P., Trieu-Cuot P. (2003). Genetic Basis of Antibiotic Resistance in Streptococcus agalactiae Strains Isolated in a French Hospital. Antimicrob. Agents Chemother.

[74] Aminov R.I., Garrigues-Jeanjean N., Mackie R.I. (2001). Molecular Ecology of Tetracycline Resistance: Development and Validation of Primers for Detection of Tetracycline Resistance Genes Encoding Ribosomal Protection Proteins. Appl. Environ. Microbiol..

[75] Lanz R., Kuhnert P., Boerlin P. (2003). Antimicrobial resistance and resistance gene determinants in clinical Escherichia coli from different animal species in Switzerland. Vet. Microbiol..

[76] Call D.R., Bakko M.K., Krug M.J., Roberts M.C. (2003). Identifying Antimicrobial Resistance Genes with DNA Microarrays. Antimicrob. Agents Chemother..

[77] Iwahara K., Kuriyama T., Shimura S., Williams D.W., Yanagisawa M., Nakagawa K., Karasawa T. (2006). Detection of cfxA and cfxA2, the beta-lactamase genes of Prevotella spp., in clinical samples from dentoalveolar infection by real-time PCR. J. Clin. Microbiol..

[78] Dutour C., Bonnet R., Marchandin H., Boyer M., Chanal C., Sirot D., Sirot J. (2002). CTX-M-1, CTX-M-3, and CTX-M-14 beta-lactamases from Enterobacteriaceae isolated in France. Antimicrob. Agents Chemother..

[79] Ehrmann E., Handal T., Tamanai-Shacoori Z., Bonnaure-Mallet M., Fosse T. (2014). High prevalence of -lactam and macrolide resistance genes in human oral Capnocytophaga species. J. Antimicrob. Chemother..

[80] Voha C., Docquier J.-D., Rossolini G.M., Fosse T. (2006). Genetic and biochemical characterization of FUS-1 (OXA-85), a narrow-spectrum class D beta-lactamase from Fusobacterium nucleatum subsp. polymorphum. Antimicrob. Agents Chemother..

[81] Böckelmann U., Dörries H.-H., Ayuso-Gabella M.N., Salgot de Marçay M., Tandoi V., Levantesi C., Masciopinto C., van Houtte E., Szewzyk U., Wintgens T. (2009). Quantitative PCR Monitoring of Antibiotic Resistance Genes and Bacterial Pathogens in Three European Artificial Groundwater Recharge Systems. Appl. Environ. Microbiol..

[82] Malhotra-Kumar S., Lammens C., Piessens J., Goossens H. (2005). Multiplex PCR for Simultaneous Detection of Macrolide and Tetracycline Resistance Determinants in Streptococci. Antimicrob. Agents Chemother..

[83] Perreten V., Vorlet-Fawer L., Slickers P., Ehricht R., Kuhnert P., Frey J. (2005). Microarray-Based Detection of 90 Antibiotic Resistance Genes of Gram-Positive Bacteria. J. Clin. Microbiol..

[84] Kangaba A.A., Saglam F.Y., Tokman H.B., Torun M., Torun M.M. (2015). The prevalence of enterotoxin and antibiotic resistance genes in clinical and intestinal Bacteroides fragilis group isolates in Turkey. Anaerobe.

[85] Daly M.M., Doktor S., Flamm R., Shortridge D. (2004). Characterization and prevalence of MefA, MefE, and the associated msr(D) gene in Streptococcus pneumoniae clinical isolates. J. Clin. Microbiol..

[86] Sutcliffe J., Grebe T., Tait-Kamradt A., Wondrack L. (1996). Detection of erythromycin-resistant determinants by PCR. Antimicrob. Agents Chemother..

[87] Dutka-Malen S., Evers S., Courvalin P. (1995). Detection of glycopeptide resistance genotypes and identification to the species level of clinically relevant enterococci by PCR. J. Clin. Microbiol..

[88] Depardieu F., Perichon B., Courvalin P. (2004). Detection of the van Alphabet and Identification of Enterococci and Staphylococci at the Species Level by Multiplex PCR. J. Clin. Microbiol..

[89] Fines M., Perichon B., Reynolds P., Sahm D.F., Courvalin P. (1999). VanE, a New Type of Acquired Glycopeptide Resistance in Enterococcus faecalis BM4405. Antimicrob. Agents Chemother..

[90] Rebelo A.R., Bortolaia V., Kjeldgaard J.S., Pedersen S.K., Leekitcharoenphon P., Hansen I.M., Guerra B., Malorny B., Borowiak M., Hammerl J.A. (2018). Multiplex PCR for detection of plasmid-mediated colistin resistance determinants, mcr-1, mcr-2, mcr-3, mcr-4 and mcr-5 for surveillance purposes. Eurosurveillance.

[91] Malbruny B., Werno A.M., Murdoch D.R., Leclercq R., Cattoir V. (2011). Cross-Resistance to Lincosamides, Streptogramins A, and Pleuromutilins Due to the lsa (C) Gene in Streptococcus agalactiae UCN70. Antimicrob. Agents Chemother..

[92] Navas J., Fernández-Martínez M., Salas C., Cano M.E., Martínez-Martínez L. (2016). Susceptibility to Aminoglycosides and Distribution of aph and aac(3)-XI Genes among Corynebacterium striatum Clinical Isolates. PLoS ONE.

[93] Anderson A.C., Sanunu M., Schneider C., Clad A., Karygianni L., Hellwig E., Al-Ahmad A. (2014). Rapid species-level identification of vaginal and oral lactobacilli using MALDI-TOF MS analysis and 16S rDNA sequencing. BMC Microbiol..

