# Supplementary material for: Phenotypic Adaptation to Antiseptics and Effects on Biofilm Formation Capacity and Antibiotic Resistance in Clinical Isolates of Early Colonizers in Dental Plaque
Source: Antibiotics (Basel). 2022 May 19;11(5):688. doi: 10.3390/antibiotics11050688 (PMC9137571; doi:10.3390/antibiotics11050688)
Supplement: Supplementary file 1 [file antibiotics-11-00688-s001.zip › antibiotics-1731344-supplementary.pdf]

Phenotypic adaptation toward antiseptics and effects on biofilm formation capacity and antibiotic resistance in clinical isolates of early colonizers in dental plaque

David L. Auer[1,#], Xiaojun Mao[1,#], Annette Carola Anderson[2], Denise Muehler[1], Annette Wittmer[3], Christiane von Ohle[4], Diana Wolff[5], Cornelia Frese[5], Karl-Anton Hiller[1], Tim Maisch[6], Wolfgang Buchalla[1], Elmar Hellwig[2], Ali Al-Ahmad[2,§] and Fabian Cieplik[1,§,*]

[1] Department of Conservative Dentistry and Periodontology, University Hospital Regensburg, Germany

[2] Department of Operative Dentistry and Periodontology, Center for Dental Medicine, Faculty of Medicine, University of Freiburg, Germany

[3] Institute of Medical Microbiology and Hygiene, Faculty of Medicine, University of Freiburg, Freiburg, Germany

[4] Department of Conservative Dentistry, Periodontology and Endodontology, University Hospital Tübingen, Germany

[5] Department of Conservative Dentistry, University Hospital Heidelberg, Germany

[6] Department of Dermatology, University Hospital Regensburg, Germany

[#] These authors contributed equally and share first authorship.

[§] These authors contributed equally and share last authorship.

*** Corresponding author:**

Priv.-Doz. Dr. Fabian Cieplik

Department of Conservative Dentistry and Periodontology, University Hospital Regensburg

Franz-Josef-Strauß-Allee 11, 93053 Regensburg, Germany

Email: fabian.cieplik@ukr.de

SUPPLEMENTARY INFORMATION

Supplementary Table S1: Primers used for the detection of antibiotic resistance genes (ARGs)

Target gene	Conferring resistance toward	Forward and reverse primers	Primer concentration	Tm [°C]	Reference
tetM	tetracyclines	tetM-f: AGTTTTAGCTCATGTTGATG<br>tetM-r: TCCGACTATTTAGACGACGG	100nM<br>100nM	55	[1]
tetO	tetracyclines	tetO-f: GCGGAACATTGCATTTGAGGG<br>tetO-r: CTCTATGGACAACCCGACAGAAG	100nM<br>100nM	53	[2]
tetW	tetracyclines	tetW-f: GAGAGCCTGCTATATGCCAGC<br>tetW-f: GGGCGTATCCACAATGTTAAC	100nM<br>100nM	64	[3]
tetA-1	tetracyclines	tetA-1-f: GGCGGTCTTCTTCATCATGC<br>tetA-1-r: CGGCAGGCAGAGCAAGTAGA	250nM<br>250nM	64	[4]
tetB-1	tetracyclines	tetB-1-f: CATTAATAGGCGCATCGCTG<br>tetB-1-r: TGAAGGTCATCGATAGCAGG	250nM<br>250nM	64	[4]
tetC-1	tetracyclines	tetC-1-f: GCTGTAGGCATAGGCTTGGT<br>tetC-1-r: GCCGGAAGCGAGAAGAATCA	250nM<br>250nM	65	[4]
tetD-1	tetracyclines	tetD-1-f: GCAAACCATTACGGCATTCT<br>tetD-1-r: GATAAGCTGCGCGGTAAAAA	400nM<br>400nM	60	[5]
tetE-1	tetracyclines	tetE-1-f: TATTAACGGGCTGGCATTTC<br>tetE-1-r: AGCTGTCAGGTGGGTCAAAC	400nM<br>400nM	60	[5]
bla_{TEM-1}	β-lactams	bla_{TEM-1}-f: CCAATGCTTAATCAGTGAGG<br>bla_{TEM-1}-r: ATGAGTATTCAACATTTCCG	400nM<br>400nM	60	[5]
cfxA	cephamycins	cfxA-f: GCAAGTGCAGTTTAAGATT<br>cfxA-f: GCTTTAGTTTGCATTTTCATC	200nM<br>200nM	58	[6]
$bla_{CTX-M-1}$	cephalosporins	CTX-M-f: CGCTTTGCGATGTGCAG<br>CTX-M-f: ACCGCGATATCGTTGGT	100nM<br>100nM	56	[7]
bla_{CSP-1}	β-lactams	bla_{CSP-1}-f: TCAAATTTCACATTGCATTG<br>bla_{CSP-1}-f: CAGTGTGGGATTATTATTTTCA	100nM<br>100nM	52	[8]
bla_{OXA-85}	β-lactams	bla_{OXA-85}-f: CCATATGTTATTATTTATGTTCTCGAT<br>bla_{OXA-85}-f: GCGGATCCTTATTTTATAACATTTATATTTTTG	1µM<br>1µM	52	[9]
ampC	ampicillin	ampC-f: GTGACCAGATACTGGCCACA<br>ampC-r: TTACTGTAGCGCCTCGAGGA	100nM<br>100nM	56	[10]
pbpX2	penams, cephamycins, cephalosporins	pbpX2-f: GTATATGACCGCGACCTTGG<br>pbpX2-r: TTCTTGATGCTAGGCATTGC	200nM<br>200nM	54	[11]
ermA	erythromycin	ermA-f: CCCGAAAAATACGCAAAATTTCAT<br>ermA-r: CCCTGTTTACCCATTTATAAACG	500nM<br>500nM	62	[12]
ermB	erythromycin	ermB-f: GAAAAGGTACTCAACCAAATA<br>ermB-r: AGTAACGGTACTTAAATTGTTTAC	100nM<br>100nM	52	[13]
ermC	erythromycin	ermC-f: AATCGGCTCAGGAAAAGG<br>ermC-r: ATCGTCAATTCCTGCATG	100nM<br>100nM	54	[14]
ermF	erythromycin	ermF-f: CCT TAT GGC ATT ACT TCC GA<br>ermF-r: GGA CCT ACC TCA TAG ACA AG	200nM<br>200nM	57	[15]
MefI	macrolides	MefI-f: ATGGAAAAATACAACAATTGGAAA<br>MefI-r: CCAGCTGCTGCGATAATTAA	100nM<br>100nM	52	[13]
mefAI	macrolides	mefAI-f: TGGTTCGGTGCTTACTATTGT<br>mefAI-r: CCCCTATCAACATTCCAGA	100nM<br>100nM	52	[16]
mefAII	macrolides	mefAII-f: AGTATCATTAATCACTAGTGC<br>mefAII-r: TTCTTCTGGTACTAAAAGTGG	100nM<br>100nM	53	[17]
patA	fluoroquinolones	patA-f: ATGTTGTCCTCGCAGCCTAT<br>patA-r: ACGAACCGATGAACAAGAGG	100nM<br>100nM	47	[18]
patB	fluoroquinolones	patB-f: TTGCTGGTTCGGCTGTACTT<br>patB-r: AACTGCTGTCATCTGGCCTT	100nM<br>100nM	47	[18]

vanA	vancomycin	vanA-f: GGGAAAACGACAATTGC<br>vanA-r: GTACAATGCGGCCGTTA	200nM<br>200nM	54	[19]
vanB	vancomycin	vanB-f: ATGGGAAGCCGATAGTC<br>vanB-r: GATTTCGTTCCTCGACC	200nM<br>200nM	54	[19]
vanC1	vancomycin	vanC1-f: GGTATCAAGGAAACCTC<br>vanC1-r: CTTCCGCCATCATAGCT	500nM<br>500nM	50	[1]
vanC2/3	vancomycin	vanC2/3-f: CTCCTACGATTCTCTTG<br>vanC2/3-r: CGAGCAAGACCTTTAAG	500nM<br>500nM	50	[1]
vanD	vancomycin	vanD-f: TGTGGGATGCGATATTTCAA<br>vanD-r: TGCAGCCAAGTATCCGGTAA	320nM<br>320nM	54	[20]
vanE	vancomycin	vanD-f: TGTGGGATGCGATATTTCAA<br>vanD-r: TGCAGCCAAGTATCCGGTAA	500nM<br>500nM	52	[21]
mcr-1	colistin	Mcr1-f: AGTCCGTTTGTTCTTGTGGC<br>Mcr1-r: AGATCCTTGGTCTCGGCTTG	100nM<br>100nM	58	[22]
lsaC	lincosamides, streptogramines, pleuromutilines	IsaC-f: GGCTATGTAAAACCTGTATTTG<br>IsaC-r: ACTGACAATTTTTCTTCCGT	100nM<br>100nM	53	[23]
aph(3'')-Ib	amino-glycosides	aph(3'')-Ib-f: CTTGGTGATAACGGCAATTC<br>aph(3'')-Ib-r: CCAATCGCAGATAGAAGGC	400nM<br>400nM	52	[24]
int-II integrase		int-II-f: GCGTGATTGTATCTCACT<br>int-II-r: GACGCTCCTGTTGCTTCT	250nM<br>250nM	50	[25]
xis-II excisionase		xis-II-f: AAGCAGACTGACATTCCTA<br>xis-II-r: GCGTCCAATGTATCTATAA	250nM<br>250nM	50	[25]
Universal bacterial PCR as positive control (27f_YM-1492R)		27f-YM: AGAGTTTGATYMTGGCTCAG<br>1492r-long: TACGGYTACCTTGTTACGACTT	300nM<br>300nM	55	[26]

Supplementary Table S2: Detailed results from MIC passaging for all investigated clinical isolates

No.	Genus	Species	CHX						CPC					
			P1 MIC	P10 MIC	Factor to P1	Reevaluation MIC	Factor to P1	Factor to P10	P1 MIC	P10 MIC	Factor to P1	Reevaluation MIC	Factor to P1	Factor to P10
1	*Streptococcus*	*anginosus*	8	16	2	*	0	0	2	1	0.5	*	-	-
2	*Streptococcus*	*anginosus*	4	16	4	8	2	0.5	1	1	1	*	-	-
3	*Streptococcus*	*anginosus*	8	16	2	*	0	0	2	2	1	*	-	-
4	*Streptococcus*	*anginosus*	8	8	1	*	0	0	2	2	1	*	-	-
5	*Streptococcus*	*anginosus*	8	16	2	*	0	0	4	2	0.5	*	-	-
6	*Streptococcus*	*anginosus*	8	32	4	16	2	0.5	4	2	0.5	*	-	-
7	*Streptococcus*	*anginosus*	8	16	2	*	0	0	4	2	0.5	*	-	-
8	*Streptococcus*	*anginosus*	8	16	2	*	0	0	1	2	2	*	-	-
9	*Streptococcus*	*anginosus*	8	16	2	*	0	0	4	2	0.5	*	-	-
10	*Streptococcus*	*anginosus*	2	8	4	16	8	2	2	2	1	*	-	-
11	*Streptococcus*	*anginosus*	4	16	4	16	4	1	2	2	1	*	-	-
12	*Streptococcus*	*constellatus*	4	4	1	*	0	0	1	2	2	*	-	-
13	*Streptococcus*	*oralis*	2	8	4	32	16	4	1	1	1	*	-	-
14	*Streptococcus*	*oralis*	4	16	4	32	8	2	4	2	0.5	*	-	-
15	*Streptococcus*	*oralis*	4	16	4	16	4	1	1	1	1	*	-	-
16	*Streptococcus*	*oralis*	4	16	4	16	4	1	1	1	1	*	-	-
17	*Streptococcus*	*oralis*	8	16	2	*	0	0	1	1	1	*	-	-
18	*Streptococcus*	*oralis*	8	16	2	*	0	0	1	2	2	*	-	-
19	*Streptococcus*	*oralis*	4	8	2	*	0	0	2	2	1	*	-	-
20	*Streptococcus*	*oralis*	8	16	2	*	0	0	2	4	2	*	-	-
21	*Streptococcus*	*oralis*	8	16	2	*	0	0	2	2	1	*	-	-
22	*Streptococcus*	*oralis*	8	16	2	*	0	0	2	2	1	*	-	-

23	*Streptococcus*	*oralis*	16	16	1	*	0	0	2	2	1	*	-	-
24	*Streptococcus*	*oralis*	16	8	0.5	*	0	0	2	2	1	*	-	-
25	*Streptococcus*	*oralis*	8	8	1	*	0	0	2	2	1	*	-	-
27	*Streptococcus*	*oralis*	16	16	1	*	0	0	2	2	1	*	-	-
28	*Streptococcus*	*oralis*	8	16	2	*	0	0	2	4	2	*	-	-
29	*Streptococcus*	*oralis*	4	16	4	16	4	1	4	4	1	*	-	-
30	*Streptococcus*	*oralis*	32	16	0.5	*	0	0	2	8	4	2	1	0.25
31	*Streptococcus*	*oralis*	16	16	1	*	0	0	2	2	1	*	-	-
32	*Streptococcus*	*oralis*	32	16	0.5	*	0	0	2	2	1	*	-	-
33	*Streptococcus*	*oralis*	16	16	1	*	0	0	2	4	2	*	-	-
34	*Streptococcus*	*oralis*	16	16	1	*	0	0	2	4	2	*	-	-
35	*Streptococcus*	*oralis*	32	16	0.5	*	0	0	2	2	1	*	-	-
36	*Streptococcus*	*oralis*	32	16	0.5	*	0	0	2	2	1	*	-	-
37	*Streptococcus*	*sanguinis*	16	8	0.5	*	0	0	2	4	2	*	-	-
38	*Streptococcus*	*sanguinis*	8	16	2	*	0	0	2	4	2	*	-	-
39	*Streptococcus*	*sanguinis*	8	8	1	*	0	0	4	4	1	*	-	-
40	*Streptococcus*	*sanguinis*	16	8	0.5	*	0	0	4	4	1	*	-	-
41	*Streptococcus*	*sanguinis*	16	16	1	*	0	0	4	2	0.5	*	-	-
42	*Streptococcus*	*sanguinis*	32	16	0.5	*	0	0	4	16	4	8	2	0.5
43	*Streptococcus*	*sanguinis*	32	32	1	*	0	0	4	2	0.5	*	-	-
44	*Streptococcus*	*sanguinis*	32	16	0.5	*	0	0	2	2	1	*	-	-
45	*Streptococcus*	*sanguinis*	8	16	2	*	0	0	2	4	2	*	-	-
46	*Streptococcus*	*sanguinis*	8	16	2	*	0	0	4	2	0.5	*	-	-
47	*Streptococcus*	*sanguinis*	16	32	2	*	0	0	8	2	0.25	*	-	-
48	*Streptococcus*	*sanguinis*	32	16	0.5	*	0	0	8	2	0.25	*	-	-
49	*Streptococcus*	*sanguinis*	32	16	0.5	*	0	0	8	2	0.25	*	-	-
50	*Streptococcus*	*sanguinis*	4	8	2	*	0	0	4	2	0.5	*	-	-
51	*Streptococcus*	*sanguinis*	2	8	4	8	4	1	8	2	0.25	*	-	-
52	*Streptococcus*	*sanguinis*	4	4	1	*	0	0	4	2	0.5	*	-	-
53	*Streptococcus*	*sanguinis*	4	4	1	*	0	0	4	2	0.5	*	-	-

56	*Streptococcus*	*sanguinis*	4	4	1	*	0	0	4	16	4	8	2	0.5
57	*Streptococcus*	*intermedius*	4	8	2	*	0	0	8	4	0.5	*	-	-
59	*Streptococcus*	*mutans*	0.5	4	8	4	8	1	8	2	0.25	*	-	-
60	*Streptococcus*	*gordonii*	8	8	1	*	0	0	8	4	0.5	*	-	-
61	*Streptococcus*	*gordonii*	8	16	2	*	0	0	2	4	2	*	-	-
62	*Streptococcus*	*gordonii*	4	16	4	32	8	2	2	4	2	*	-	-
63	*Streptococcus*	*gordonii*	8	16	2	*	0	0	4	1	0.25	*	-	-
64	*Streptococcus*	*gordonii*	4	16	4	32	8	2	2	1	0.5	*	-	-
65	*Streptococcus*	*gordonii*	4	16	4	16	4	1	2	4	2	*	-	-
66	*Streptococcus*	*gordonii*	8	8	1	*	0	0	2	4	2	*	-	-
67	*Streptococcus*	*gordonii*	4	8	2	*	0	0	4	4	1	*	-	-
68	*Streptococcus*	*gordonii*	4	16	4	16	4	1	4	2	0.5	*	-	-
69	*Streptococcus*	*salivarius*	2	8	4	8	4	1	2	2	1	*	-	-
70	*Streptococcus*	*salivarius*	4	8	2	*	0	0	2	2	1	*	-	-
73	*Streptococcus*	*salivarius*	1	8	8	8	8	1	2	2	1	*	-	-
74	*Streptococcus*	*salivarius*	4	16	4	16	4	1	2	2	1	*	-	-
75	*Streptococcus*	*salivarius*	4	4	1	*	0	0	2	2	1	*	-	-
76	*Streptococcus*	*salivarius*	4	2	0.5	*	0	0	2	2	1	*	-	-
77	*Streptococcus*	*sobrinus*	4	4	1	*	0	0	2	2	1	*	-	-
78	*Streptococcus*	*vestibularis*	1	8	8	8	8	1	2	1	0.5	*	-	-
80	*Streptococcus*	*downii*	4	16	4	16	4	1	4	1	0.25	*	-	-
81	*Streptococcus*	*downii*	4	16	4	16	4	1	2	1	0.5	*	-	-
82	*Streptococcus*	*parasanguinis*	4	8	2	*	0	0	4	2	0.5	*	-	-
83	*Streptococcus*	*parasanguinis*	8	16	2	*	0	0	2	2	1	*	-	-
84	*Streptococcus*	*parasanguinis*	4	16	4	32	8	2	4	2	0.5	*	-	-
85	*Streptococcus*	*parasanguinis*	4	16	4	16	4	1	4	2	0.5	*	-	-
86	*Streptococcus*	*parasanguinis*	4	16	4	16	4	1	4	2	0.5	*	-	-
87	*Streptococcus*	*parasanguinis*	8	16	2	*	0	0	2	2	1	*	-	-
89	*Streptococcus*	*mitis*	4	4	1	*	0	0	2	2	1	*	-	-
90	*Streptococcus*	*oralis*	4	16	4	32	8	2	4	2	0.5	*	-	-

91	*Streptococcus*	*mitis*	2	4	2	*	0	0	1	2	2	*	-	-
92	*Streptococcus*	*mitis*	2	4	2	*	0	0	1	2	2	*	-	-
93	*Streptococcus*	*mitis*	1	8	8	8	8	1	1	1	1	*	-	-
94	*Streptococcus*	*mitis*	2	8	4	4	2	0.5	1	2	2	*	-	-
95	*Streptococcus*	*mitis*	2	4	2	*	0	0	1	2	2	*	-	-
96	*Streptococcus*	*mitis*	2	8	4	8	4	1	1	1	1	*	-	-
97	*Streptococcus*	*mitis*	4	8	2	*	0	0	0.5	2	4	*	-	-
98	*Streptococcus*	*mitis*	4	8	2	*	0	0	1	2	2	*	-	-
99	*Streptococcus*	*mitis*	4	8	2	*	0	0	1	2	2	*	-	-
100	*Streptococcus*	*mitis*	4	8	2	*	0	0	1	2	2	*	-	-
101	*Streptococcus*	*mutans*	4	4	1	*	0	0	2	4	2	*	-	-
102	*Streptococcus*	*mutans*	4	8	2	*	0	0	2	4	2	*	-	-
103	*Streptococcus*	*mutans*	4	4	1	*	0	0	2	4	2	*	-	-
104	*Streptococcus*	*mutans*	4	4	1	*	0	0	2	1	0.5	*	-	-
105	*Streptococcus*	*mutans*	4	8	2	*	0	0	2	4	2	*	-	-
106	*Streptococcus*	*mutans*	8	4	0.5	*	0	0	2	4	2	*	-	-
107	*Streptococcus*	*mutans*	4	4	1	*	0	0	2	2	1	*	-	-
108	*Streptococcus*	*mutans*	4	4	1	*	0	0	2	4	2	*	-	-
109	*Streptococcus*	*mutans*	4	4	1	*	0	0	2	8	4	2	1	0.25
110	*Streptococcus*	*mutans*	8	8	1	*	0	0	2	4	2	*	-	-
111	*Streptococcus*	*mutans*	4	4	1	*	0	0	2	2	1	*	-	-
112	*Streptococcus*	*mutans*	4	4	1	*	0	0	2	2	1	*	-	-
113	*Streptococcus*	*mutans*	4	4	1	*	0	0	2	2	1	*	-	-
114	*Streptococcus*	*mutans*	4	4	1	*	0	0	2	4	2	*	-	-
115	*Streptococcus*	*mutans*	4	4	1	*	0	0	2	4	2	*	-	-
116	*Streptococcus*	*mutans*	4	8	2	*	0	0	2	2	1	*	-	-
117	*Streptococcus*	*mutans*	4	4	1	*	0	0	2	2	1	*	-	-
118	*Streptococcus*	*cristatus*	4	8	2	*	0	0	2	4	2	*	-	-
119	*Streptococcus*	*cristatus*	2	4	2	*	0	0	1	4	4	8	8	2
120	*Streptococcus*	*cristatus*	2	4	2	*	0	0	1	1	1	*	-	-

201	*Actinomyces*	*naeslundii*	2	2	1	*	-	-	1	2	2	*	-	-
202	*Actinomyces*	*naeslundii*	1	2	2	*	-	-	1	2	2	*	-	-
203	*Actinomyces*	*naeslundii*	2	2	1	*	-	-	2	2	1	*	-	-
204	*Actinomyces*	*naeslundii*	1	2	2	*	-	-	2	1	0,5	*	-	-
205	*Actinomyces*	*naeslundii*	2	2	1	*	-	-	2	2	1	*	-	-
206	*Actinomyces*	*naeslundii*	0.5	1	2	*	-	-	2	2	1	*	-	-
207	*Actinomyces*	*naeslundii*	0.5	1	2	*	-	-	2	2	1	*	-	-
208	*Actinomyces*	*oris*	2	2	1	*	-	-	2	2	1	*	-	-
209	*Actinomyces*	*oris*	0.5	2	4	*	-	-	4	2	0,5	*	-	-
210	*Actinomyces*	*oris*	2	2	1	*	-	-	4	4	1	*	-	-
211	*Actinomyces*	*oris*	4	4	1	*	-	-	4	2	0,5	*	-	-
212	*Actinomyces*	*oris*	1	4	4	*	-	-	4	2	0,5	*	-	-
213	*Actinomyces*	*odontolyticus*	4	4	1	*	-	-	4	2	0,5	*	-	-
214	*Actinomyces*	*odontolyticus*	4	4	1	*	-	-	4	2	0,5	*	-	-
215	*Actinomyces*	*odontolyticus*	8	4	0.5	*	-	-	4	2	0,5	*	-	-
216	*Actinomyces*	*odontolyticus*	4	2	0.5	*	-	-	4	2	0,5	*	-	-
217	*Actinomyces*	*odontolyticus*	4	4	1	*	-	-	4	2	0,5	*	-	-
218	*Actinomyces*	*odontolyticus*	2	2	1	*	-	-	4	2	0,5	*	-	-
219	*Actinomyces*	*odontolyticus*	1	2	2	*	-	-	2	2	1	*	-	-
301	*Rothia*	*aeria*	2	2	1	*	-	-	4	2	0,5	*	-	-
302	*Rothia*	*aeria*	2	2	1	*	-	-	4	2	0,5	*	-	-
303	*Rothia*	*aeria*	1	2	2	*	-	-	4	2	0,5	*	-	-
304	*Rothia*	*aeria*	4	4	1	*	-	-	2	2	1	*	-	-
305	*Rothia*	*aeria*	4	8	2	*	-	-	2	2	1	*	-	-
306	*Rothia*	*aeria*	4	4	1	*	-	-	2	2	1	*	-	-
307	*Rothia*	*dentocariosa*	4	4	1	*	-	-	2	2	1	*	-	-
308	*Rothia*	*dentocariosa*	4	4	1	*	-	-	2	2	1	*	-	-

309	*Rothia*	*dentocariosa*	4	4	1	*	-	-	2	2	1	*	-	-
310	*Rothia*	*dentocariosa*	4	4	1	*	-	-	2	1	0,5	*	-	-
311	*Rothia*	*dentocariosa*	4	4	1	*	-	-	2	2	1	*	-	-
312	*Rothia*	*dentocariosa*	4	4	1	*	-	-	2	2	1	*	-	-
313	*Rothia*	*mucilaginosa*	4	8	2	*	-	-	2	2	1	*	-	-
314	*Rothia*	*mucilaginosa*	4	8	2	*	-	-	2	2	1	*	-	-
315	*Rothia*	*mucilaginosa*	4	4	1	*	-	-	2	2	1	*	-	-
316	*Rothia*	*mucilaginosa*	2	4	2	*	-	-	1	2	2	*	-	-
317	*Rothia*	*mucilaginosa*	4	4	1	*	-	-	4	2	0,5	*	-	-
318	*Rothia*	*mucilaginosa*	2	4	2	*	-	-	1	1	1	*	-	-
319	*Rothia*	*mucilaginosa*	4	4	1	*	-	-	1	2	2	*	-	-
320	*Rothia*	*aeria*	2	4	2	*	-	-	1	2	2	*	-	-
401	*Veillonella*	*atypica*	1	2	2	*	-	-	1	2	2	*	-	-
402	*Veillonella*	*atypica*	1	2	2	*	-	-	1	2	2	*	-	-
403	*Veillonella*	*atypica*	1	1	1	*	-	-	1	2	2	*	-	-
404	*Veillonella*	*atypica*	1	2	2	*	-	-	1	1	1	*	-	-
405	*Veillonella*	*atypica*	1	2	2	*	-	-	2	2	1	*	-	-
406	*Veillonella*	*atypica*	2	1	0.5	*	-	-	1	2	2	*	-	-
407	*Veillonella*	*atypica*	2	2	1	*	-	-	2	1	0.5	*	-	-
408	*Veillonella*	*atypica*	0.5	1	2	*	-	-	1	2	2	*	-	-
409	*Veillonella*	*atypica*	1	1	1	*	-	-	2	2	1	*	-	-
410	*Veillonella*	*atypica*	1	2	2	*	-	-	0.5	1	2	*	-	-
411	*Veillonella*	*dispar*	2	1	0.5	*	-	-	1	2	2	*	-	-
412	*Veillonella*	*dispar*	2	1	0.5	*	-	-	0.5	2	4	4	8	2
413	*Veillonella*	*parvula*	1	1	1	*	-	-	1	2	2	*	-	-
414	*Veillonella*	*parvula*	1	2	2	*	-	-	1	2	2	*	-	-
415	*Veillonella*	*parvula*	2	2	1	*	-	-	2	4	2	*	-	-
417	*Veillonella*	*parvula*	2	2	1	*	-	-	2	2	1	*	-	-

418	*Veillonella*	*parvula*	1	2	2	*	-	-	2	2	1	*	-	-
419	*Veillonella*	*parvula*	1	1	1	*	-	-	1	2	2	*	-	-
420	*Veillonella*	*parvula*	2	1	0.5	*	-	-	1	2	2	*	-	-
421	*Veillonella*	*parvula*	1	1	1	*	-	-	2	2	1	*	-	-
422	*Veillonella*	*parvula*	2	2	1	*	-	-	1	2	2	*	-	-
423	*Veillonella*	*parvula*	2	2	1	*	-	-	2	2	1	*	-	-
424	*Veillonella*	*parvula*	1	1	1	*	-	-	1	2	2	*	-	-
425	*Veillonella*	*parvula*	2	1	0.5	*	-	-	2	2	1	*	-	-
426	*Veillonella*	*parvula*	2	2	1	*	-	-	2	1	0.5	*	-	-
427	*Veillonella*	*parvula*	2	1	0.5	*	-	-	1	2	2	*	-	-

References

1. Lins, R.X.; Oliveira Andrade, A. de; Hirata Junior, R.; Wilson, M.J.; Lewis, M.A.; Williams, D.W.; Fidel, R.A.S. Antimicrobial resistance and virulence traits of Enterococcus faecalis from primary endodontic infections. *J. Dent.* **2013**, *41*, 779–786, doi:10.1016/j.jdent.2013.07.004.
2. Poyart, C.; Jardy, L.; Quesne, G.; Berche, P.; Trieu-Cuot, P. Genetic Basis of Antibiotic Resistance in Streptococcus agalactiae Strains Isolated in a French Hospital. *Antimicrob Agents Chemother* **2003**, *47*, 794–797, doi:10.1128/AAC.47.2.794-797.2003.
3. Aminov, R.I.; Garrigues-Jeanjean, N.; Mackie, R.I. Molecular Ecology of Tetracycline Resistance: Development and Validation of Primers for Detection of Tetracycline Resistance Genes Encoding Ribosomal Protection Proteins. *Appl Environ Microbiol* **2001**, *67*, 22–32, doi:10.1128/AEM.67.1.22-32.2001.
4. Lanz, R.; Kuhnert, P.; Boerlin, P. Antimicrobial resistance and resistance gene determinants in clinical Escherichia coli from different animal species in Switzerland. *Veterinary Microbiology* **2003**, *91*, 73–84, doi:10.1016/S0378-1135(02)00263-8.
5. Call, D.R.; Bakko, M.K.; Krug, M.J.; Roberts, M.C. Identifying Antimicrobial Resistance Genes with DNA Microarrays. *Antimicrob Agents Chemother* **2003**, *47*, 3290–3295, doi:10.1128/AAC.47.10.3290-3295.2003.
6. Iwahara, K.; Kuriyama, T.; Shimura, S.; Williams, D.W.; Yanagisawa, M.; Nakagawa, K.; Karasawa, T. Detection of cfxA and cfxA2, the beta-lactamase genes of Prevotella spp., in clinical samples from dentoalveolar infection by real-time PCR. *J. Clin. Microbiol.* **2006**, *44*, 172–176, doi:10.1128/JCM.44.1.172-176.2006.
7. Dutour, C.; Bonnet, R.; Marchandin, H.; Boyer, M.; Chanal, C.; Sirot, D.; Sirot, J. CTX-M-1, CTX-M-3, and CTX-M-14 beta-lactamases from Enterobacteriaceae isolated in France. *Antimicrob. Agents Chemother.* **2002**, *46*, 534–537, doi:10.1128/AAC.46.2.534-537.2002.
8. Ehrmann, E.; Handal, T.; Tamanai-Shacoori, Z.; Bonnaure-Mallet, M.; Fosse, T. High prevalence of -lactam and macrolide resistance genes in human oral Capnocytophaga species. *Journal of Antimicrobial Chemotherapy* **2014**, *69*, 381–384, doi:10.1093/jac/dkt350.
9. Voha, C.; Docquier, J.-D.; Rossolini, G.M.; Fosse, T. Genetic and biochemical characterization of FUS-1 (OXA-85), a narrow-spectrum class D beta-lactamase from Fusobacterium nucleatum subsp. polymorphum. *Antimicrob. Agents Chemother.* **2006**, *50*, 2673–2679, doi:10.1128/AAC.00058-06.
10. Böckelmann, U.; Dörries, H.-H.; Ayuso-Gabella, M.N.; Salgot de Marçay, M.; Tandoi, V.; Levantesi, C.; Masciopinto, C.; van Houtte, E.; Szewzyk, U.; Wintgens, T.; et al. Quantitative PCR Monitoring of Antibiotic Resistance Genes and Bacterial Pathogens in Three European Artificial Groundwater Recharge Systems. *Appl Environ Microbiol* **2009**, *75*, 154–163, doi:10.1128/AEM.01649-08.
11. Nakayama, A.; Takao, A. β-Lactam resistance in Streptococcus mitis isolated from saliva of healthy subjects. *Journal of Infection and Chemotherapy* **2003**, *9*, 321–327, doi:10.1007/s10156-003-0286-Y.
12. Malhotra-Kumar, S.; Lammens, C.; Piessens, J.; Goossens, H. Multiplex PCR for Simultaneous Detection of Macrolide and Tetracycline Resistance Determinants in Streptococci. *Antimicrob Agents Chemother* **2005**, *49*, 4798–4800, doi:10.1128/AAC.49.11.4798-4800.2005.
13. Reinert, R.R.; Filimonova, O.Y.; Al-Lahham, A.; Grudinina, S.A.; Ilina, E.N.; Weigel, L.M.; Sidorenko, S.V. Mechanisms of Macrolide Resistance among Streptococcus pneumoniae Isolates from Russia. *Antimicrob Agents Chemother* **2008**, *52*, 2260–2262, doi:10.1128/AAC.01270-07.

14. Perreten, V.; Vorlet-Fawer, L.; Slickers, P.; Ehricht, R.; Kuhnert, P.; Frey, J. Microarray-Based Detection of 90 Antibiotic Resistance Genes of Gram-Positive Bacteria. *J Clin Microbiol* **2005**, *43*, 2291–2302, doi:10.1128/JCM.43.5.2291-2302.2005.
15. Kangaba, A.A.; Saglam, F.Y.; Tokman, H.B.; Torun, M.; Torun, M.M. The prevalence of enterotoxin and antibiotic resistance genes in clinical and intestinal Bacteroides fragilis group isolates in Turkey. *Anaerobe* **2015**, *35*, 72–76, doi:10.1016/j.anaerobe.2015.07.008.
16. Daly, M.M.; Doktor, S.; Flamm, R.; Shortridge, D. Characterization and prevalence of MefA, MefE, and the associated msr(D) gene in Streptococcus pneumoniae clinical isolates. *J Clin Microbiol* **2004**, *42*, 3570–3574, doi:10.1128/JCM.42.8.3570-3574.2004.
17. Sutcliffe, J.; Grebe, T.; Tait-Kamradt, A.; Wondrack, L. Detection of erythromycin-resistant determinants by PCR. *Antimicrob Agents Chemother* **1996**, *40*, 2562–2566, doi:10.1128/AAC.40.11.2562.
18. Garvey, M.I.; Baylay, A.J.; Wong, R.L.; Piddock, L.J.V. Overexpression of patA and patB, which encode ABC transporters, is associated with fluoroquinolone resistance in clinical isolates of Streptococcus pneumoniae. *Antimicrob Agents Chemother* **2011**, *55*, 190–196, doi:10.1128/AAC.00672-10.
19. Dutka-Malen, S.; Evers, S.; Courvalin, P. Detection of glycopeptide resistance genotypes and identification to the species level of clinically relevant enterococci by PCR. *J Clin Microbiol* **1995**, *33*, 24–27, doi:10.1128/jcm.33.1.24-27.1995.
20. Depardieu, F.; Perichon, B.; Courvalin, P. Detection of the van Alphabet and Identification of Enterococci and Staphylococci at the Species Level by Multiplex PCR. *J Clin Microbiol* **2004**, *42*, 5857–5860, doi:10.1128/JCM.42.12.5857-5860.2004.
21. Fines, M.; Perichon, B.; Reynolds, P.; Sahm, D.F.; Courvalin, P. VanE, a New Type of Acquired Glycopeptide Resistance in Enterococcus faecalis BM4405. *Antimicrob Agents Chemother* **1999**, *43*, 2161–2164, doi:10.1128/AAC.43.9.2161.
22. Rebelo, A.R.; Bortolaia, V.; Kjeldgaard, J.S.; Pedersen, S.K.; Leekitcharoenphon, P.; Hansen, I.M.; Guerra, B.; Malorny, B.; Borowiak, M.; Hammerl, J.A.; et al. Multiplex PCR for detection of plasmid-mediated colistin resistance determinants, mcr-1, mcr-2, mcr-3, mcr-4 and mcr-5 for surveillance purposes. *Eurosurveillance* **2018**, *23*, doi:10.2807/1560-7917.ES.2018.23.6.17-00672.
23. Malbruny, B.; Werno, A.M.; Murdoch, D.R.; Leclercq, R.; Cattoir, V. Cross-Resistance to Lincosamides, Streptogramins A, and Pleuromutilins Due to the lsa (C) Gene in Streptococcus agalactiae UCN70. *Antimicrob. Agents Chemother.* **2011**, *55*, 1470–1474, doi:10.1128/AAC.01068-10.
24. Navas, J.; Fernández-Martínez, M.; Salas, C.; Cano, M.E.; Martínez-Martínez, L. Susceptibility to Aminoglycosides and Distribution of aph and aac(3)-XI Genes among Corynebacterium striatum Clinical Isolates. *PLoS ONE* **2016**, *11*, e0167856, doi:10.1371/journal.pone.0167856.
25. Calatayud, L.; Ardanuy, C.; Cercenado, E.; Fenoll, A.; Bouza, E.; Pallares, R.; Martín, R.; Liñares, J. Serotypes, Clones, and Mechanisms of Resistance of Erythromycin-Resistant Streptococcus pneumoniae Isolates Collected in Spain. *Antimicrob Agents Chemother* **2007**, *51*, 3240–3246, doi:10.1128/AAC.00157-07.
26. Anderson, A.C.; Sanunu, M.; Schneider, C.; Clad, A.; Karygianni, L.; Hellwig, E.; Al-Ahmad, A. Rapid species-level identification of vaginal and oral lactobacilli using MALDI-TOF MS analysis and 16S rDNA sequencing. *BMC Microbiol* **2014**, *14*, 312, doi:10.1186/s12866-014-0312-5.
